# Sources of variation in social tolerance in mouse lemurs (*Microcebus* spp.)

**DOI:** 10.1186/s12898-019-0236-x

**Published:** 2019-05-17

**Authors:** Mamy Rina Evasoa, Elke Zimmermann, Alida Frankline Hasiniaina, Solofonirina Rasoloharijaona, Blanchard Randrianambinina, Ute Radespiel

**Affiliations:** 1 Institute of Zoology, University of Veterinary Medicine Hannover, Bünteweg 17, 30559 Hannover, Germany; 2 Faculty of Sciences, University of Mahajanga, P.O. Box 652, 401 Mahajanga, Madagascar

**Keywords:** *Microcebus myoxinus*, *Microcebus ravelobensis*, *Microcebus bongolavensis*, *Microcebus danfossi*, *Microcebus margotmarshae*, *Microcebus mamiratra*, Affiliation, Female dominance, Aggression, Social encounter experiment

## Abstract

**Background:**

Social tolerance strongly influences the patterns of affiliation and aggression in animal societies. However, not much is known about the variation of social tolerance in species living in dispersed social systems that combine solitary foraging activities with the need of coordinating social interactions with conspecifics on a regular basis. This study aims to investigate the sources of variation in social tolerance within a Malagasy primate radiation with dispersed social systems, the mouse lemurs (*Microcebus* spp.). Six mouse lemur species were selected as model species that belong to three different taxonomic clades, live in two types of forest environments (dry and humid), and differed in this study with respect to their reproductive activity. Six male–female and six male–male dyads of each species were tested temporarily in a standardized social encounter paradigm in Madagascar to collect data on joint use of space, non-agonistic body contacts, aggression rates, the number of conflicts and the establishment of intra- and intersexual dominance.

**Results:**

Male–female dyads of the six species differed significantly in the frequency of affiliative and agonistic behaviors. In contrast, the variations between male–male dyads could not be explained by one parameter only, but clade membership, forest type, reproductive state as well as species were all suggested to be partially influential. Only one species (*Microcebus mamiratra*) showed signals of unambiguous female dominance in all male–female dyads, whereas the others had no or only a few dyads with female dominance.

**Conclusions:**

Variations in social tolerance and its consequences are most likely influenced by two factors, ecology (via forest type) and physiology (via reproductive activity), and only to a lesser extent by clade membership. The study suggests that mouse lemur females have higher aggression rates and more agonistic conflicts with males when females in the population are reproducing, at least in resource-rich humid forests. The study confirms a high degree of social plasticity between species in these small solitary foragers that supports their taxonomic distinctiveness and requires further scientific attention.

**Electronic supplementary material:**

The online version of this article (10.1186/s12898-019-0236-x) contains supplementary material, which is available to authorized users.

## Background

Social relationships are generally described through patterns of social interactions between individuals and form the central element of the social structure of a species [1]. Social relationships are governed by variable degrees of social tolerance that is reflected in the patterns of affiliation and aggression that can be observed between individuals [2–4]. Variations in social tolerance can affect various fitness-relevant parameters such as access to resources [5] or the selectivity and intensity of cooperation with conspecifics [6, 7]. Social tolerance levels in non-human primates have been described to differ largely between tolerant/egalitarian to intolerant/despotic societies, and much attention has already been given to this categorization, for example, in various diurnal group-living primates such as macaques [5, 8]. However, many species of primates do not live in cohesive social groups, but form dispersed social systems that are based on solitary foraging activities, and may include the formation of stable sleeping groups during periods of inactivity [9–14]. Within these systems, social interactions still occur on a regular basis, since these species are only rarely strictly territorial and therefore do meet conspecifics regularly within their home range. During such encounters, a certain level of social tolerance should be advantageous, as solitary foragers also need to coordinate various activities, such as matings [15–18], sleeping group reunions [19], access to resources when meeting at a food source that may or may not be monopolized [3, 20], coordinated movements or space use [19, 21] or predator avoidance [22]. Despite its importance, social tolerance is much less studied in small nocturnal solitary foragers due to their small size, nocturnal activity pattern and the associated difficulty to observe social encounters in dense forest environments [12].

Fichtel et al. [3] recently discriminated between two different approaches to study social tolerance that was defined as “concept that captures the probability that individuals will be in proximity to conspecifics around valuable resources with little or no aggression” [23]. The first approach investigates the “underlying behavioral traits” of social tolerance, such as non-agonistic social contacts or proximity. The second approach rather quantifies the consequences of social tolerance levels displayed in an experimentally induced competitive situation, and such studies typically analyze agonistic behaviors, aggression rates and conflict outcome. The study presented here is combining both approaches in a single design by studying for the first time social tolerance (inferred from proximity and non-agonistic contacts) AND the consequences of social tolerance (inferred from aggression rates, conflict numbers and social dominance) with a standardized experimental social encounter paradigm [24–26] that is applied with a comparative perspective to six species within a single primate radiation, mouse lemurs (*Microcebus* spp.). For five of these species, there is so far no information available on social structure, neither on its components, social interactions and relationships, nor on social tolerance and social dominance.

Mouse lemurs are nocturnal lemurs and endemic to the various humid and dry forest habitats of Madagascar [27]. A total of 24 mouse lemur species have so far been described [28]. The social system of many of these species has not yet been studied, and current knowledge is largely based on the study of seven species only (*M. berthae*, *M. griseorufus*, *M. lehilahytsara*, *M. murinus*, *Microcebus ravelobensis*, *M. rufus*, *M. sambiranensis*), most of which form some kind of sleeping groups (male–male, male–female, or female–female) during daytime in a shelter, at least temporarily, and have non-exclusive, largely overlapping home ranges not only with members of the same sleeping group but also with male strangers [reviewed in 11, 13, 29, 30]. However, based on extensive nocturnal survey work that has been conducted in many locations across Madagascar, it is evident that probably all mouse lemur species live in dispersed neighborhood systems [31], as it is typically single individuals and not groups that are encountered during the night [32–35]. It is also known that mouse lemur reproduction is highly seasonal in most species with the likely exception of those that inhabit warm lowland evergreen rainforests with high productivity such as those occurring some northern parts of Madagascar, e.g., in the region of Nosy Bé [36]. Not much is known about the social relationships in these dispersed social networks from the wild, although occasional nocturnal affiliative and agonistic encounters have been observed in several species [20, 29, 31, 37–39]. However, recent work from captivity suggests that female dominance that is untypical for mammals but formerly thought to be typical for most lemur species [40, 41], may be much more variable and plastic in mouse lemurs than expected [24, 25, 42]. For example, conflict rates, the probability for females to win conflicts, and the proportion of females being dominant over males varied between species (*M. murinus*, *M. lehilahytsara*) and season (reproductive vs. non-reproductive) and furthermore depended on age and breeding experience [25, 42]. Whether this diverse, plastic and complex behavioral phenomenon is the outcome of adaptive evolutionary trajectories of different species [25, 43] or resulted from phylogenetic constraints [41], could not be clarified so far.

**Fig. 1:**
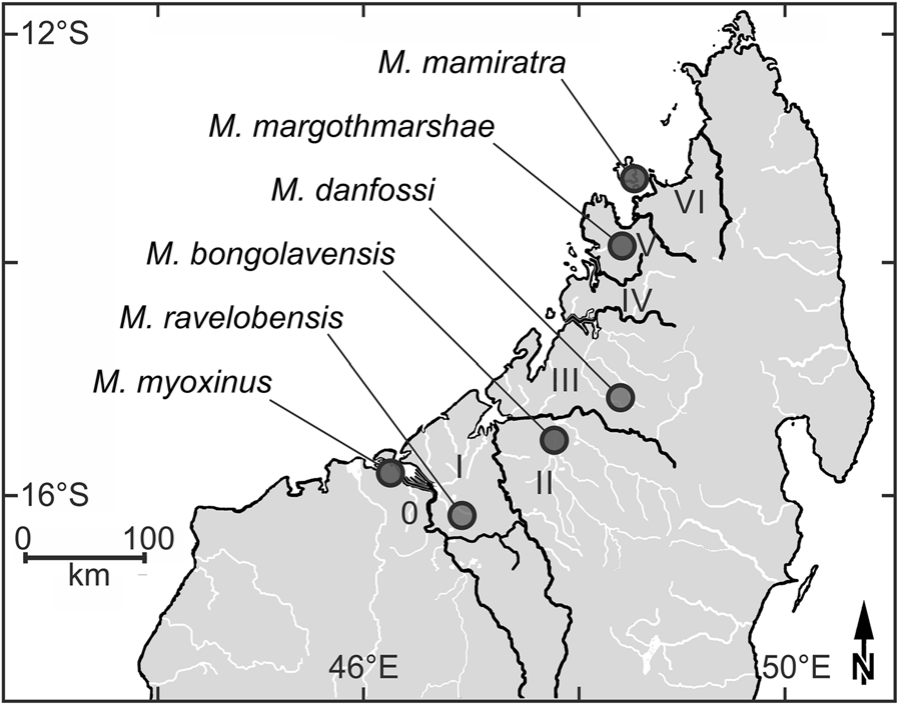
Map with northern half of Madagascar showing study sites of six study species. 0–VI: Inter-River-Systems. Geographic coordinates area provided in Additional file 1

The aim of this study is to investigate the variation in social tolerance (inferred by proximity and non-agonistic contacts) and its consequences (aggression rate, conflict numbers and social dominance) in a potentially competitive situation among six mouse lemur species (*Microcebus bongolavensis*, *Microcebus danfossi*, *Microcebus mamiratra*, *Microcebus margotmarshae*, *M. myoxinus*, *M. ravelobensis*) that have allopatric distributions along a geographic transect from northwestern to northern Madagascar (Fig. 1). These six species fall into three different phylogenetic clades with *M. mamiratra* and *M. margotmarshae* belonging to one clade (clade 1), *M. ravelobensis*, *M. bongolavensis* and *M. danfossi* forming a separate clade (clade 2), and *M. myoxinus* belonging to another clade (clade 3, Additional file 1, [28, 44]). If social tolerance is mainly influenced by phylogenetic constraints, it can be predicted that members of the same clade should show more similarities in social tolerance and its consequences than members of different phylogenetic clades.

The six species occur in two contrasting forest types, dry deciduous forests (*M. myoxinus*, *M. ravelobensis*, *M. bongolavensis*, *M. danfossi*) and low altitude humid forests (*M. margotmarshae*, *M. mamiratra*), that differ largely in the amount of yearly rainfall and the seasonality in precipitation [36]. Since rainfall has been shown to correspond to forest productivity [45, 46], these two forest types can be regarded as proxies for resource-poor (dry forest) and resource-rich conditions (humid forest) that in turn can be expected to influence social tolerance. It is predicted that the two species living in resource-rich forests (*M. margotmarshae*, *M. mamiratra*) should show higher social tolerance and less competitive tendencies (lower aggression rates and less expressed social dominance) than species living in resource-poor forests.

All species were studied in the dry season between May and October. However, not all of them were studied during the same reproductive period, since no information was available on reproduction of five of the six species at the beginning of this study. Our work then revealed that females of three species showed signals of reproductive activity (*M. danfossi*, *M. margotmarshae*, *M. mamiratra*), whereas females of the three other species showed no signal of reproduction [36]. As it is known from *M. murinus* that (1) conflict rates are higher and female dominance is more expressed in the reproductive than in the non-reproductive season [25], and that (2) males compete strongly for the access to estrous females during the reproductive season [15], it is predicted that social tolerance between males and between the sexes should be lower and competitive tendencies should be higher when estrous females are present in the population or in a dyad.

This study evaluates the potential influence of phylogeny, forest type and reproductive activity on social tolerance and its consequences in a potentially competitive situation in six mouse lemur species. We employed a standardized social encounter paradigm [24–26] that simulates biologically relevant natural encounters between unfamiliar animals which are experimentally extended in time. Due to overall time constraints, only two dyad types were tested (male–male and male–female dyads). Female–female dyads were not evaluated, because previous studies on some mouse lemur species have shown that under natural conditions females mostly interact with familiar females, i.e. related members of the same sleeping group [11, 47–49], and tend to avoid home range overlaps and social encounters with unfamiliar females of neighboring groups. Therefore, such encounter experiments do not have the same naturalistic relevance as male–male and male–female encounters which can occur at any time within home ranges.

## Results

### Variation in social tolerance among dyad types and species

The joint stay in the sleeping box, the joint use of a cage compartment, the number of total body contacts and the number of co-feeding events were analyzed to evaluate systematic variations in social tolerance between dyad partners and species.

**Fig. 2:**
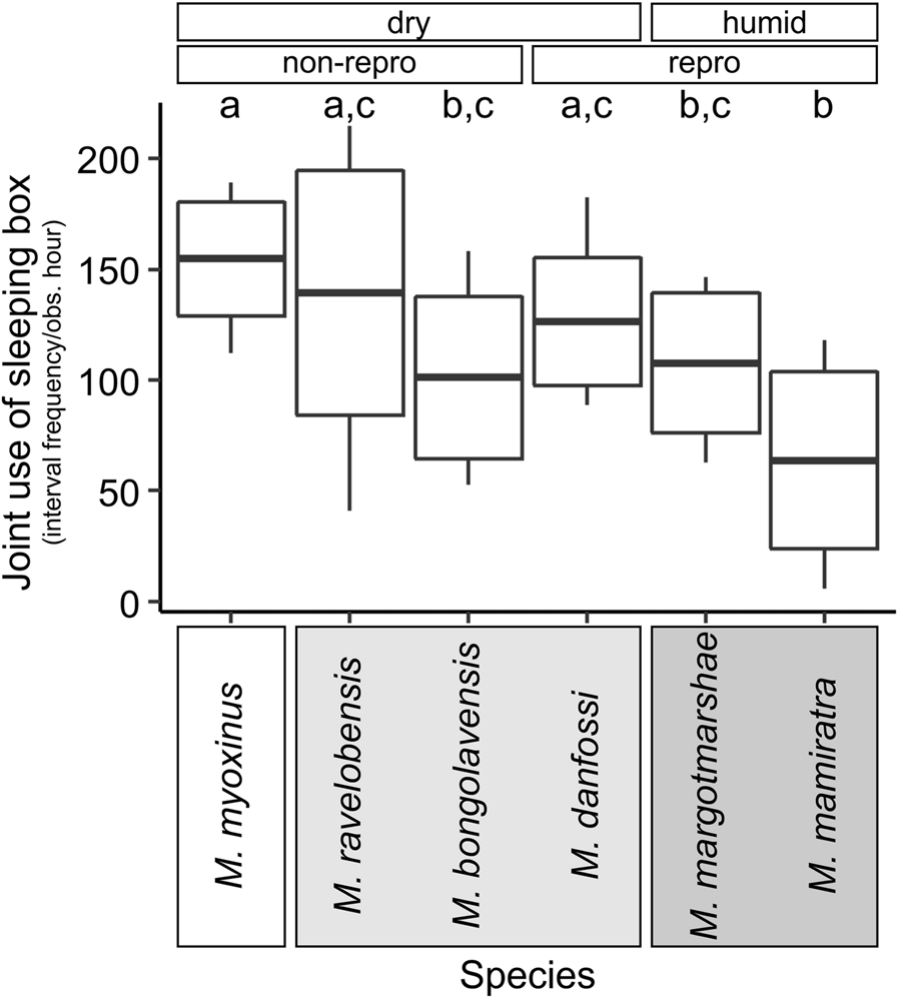
Species comparison of the *joint use of sleeping box* (interval frequency/observation hour). Mean, box: standard deviation, whiskers: minimum, maximum, Groupings according to forest type (dry, humid) and reproductive activity in the population (non-repro: no female reproductively active; repro: some females reproductively active) are indicated above the graph. Taxonomic clades (clade 3: white; clade 2: light grey; clade 1: dark grey, see Additional file 1) are indicated by boxes behind species names. Different small letters on top of box plots indicate statistical differences (p < 0.05) between species

Dyads stayed together in the sleeping box in 5.7–214.7 intervals per observation hour (1 observation hour = 240 intervals), i.e. during 2.4–89.5% of all possible intervals. The *species* model (#2) was significantly better than base model 0 (#1) at explaining the variation in the dataset, indicating that species differed significantly in the frequency of staying together in the sleeping box (Fig. 2, Additional file 2, Test 1). Although the *forest* model (#3), the *clade* model (#4), and the reproduction model (*repro*, #5) each performed significantly better than model 0 (Additional file 2, Test 1), none of them performed as well as the *species* model (#2, Additional file 2, Test 2).

The subsequent addition of the variable *pair type* did not improve the species model (#6, Additional file 2). *M. myoxinus* stayed longest together in the sleeping box and the posthoc test revealed significantly higher rates (median = 159.1 intervals/h, min = 112.2, max = 189.1) than in *M. bongolavensis*, *M. margotmarshae* and *M. mamiratra*. In contrast, *M. mamiratra* stayed shortest together in the sleeping box (median = 58.8 intervals/h, min = 5.7, max = 118.0) and had significantly lower rates than *M. myoxinus*, *M. ravelobensis* and *M. danfossi* (Fig. 2, Additional file 2).

**Fig. 3:**
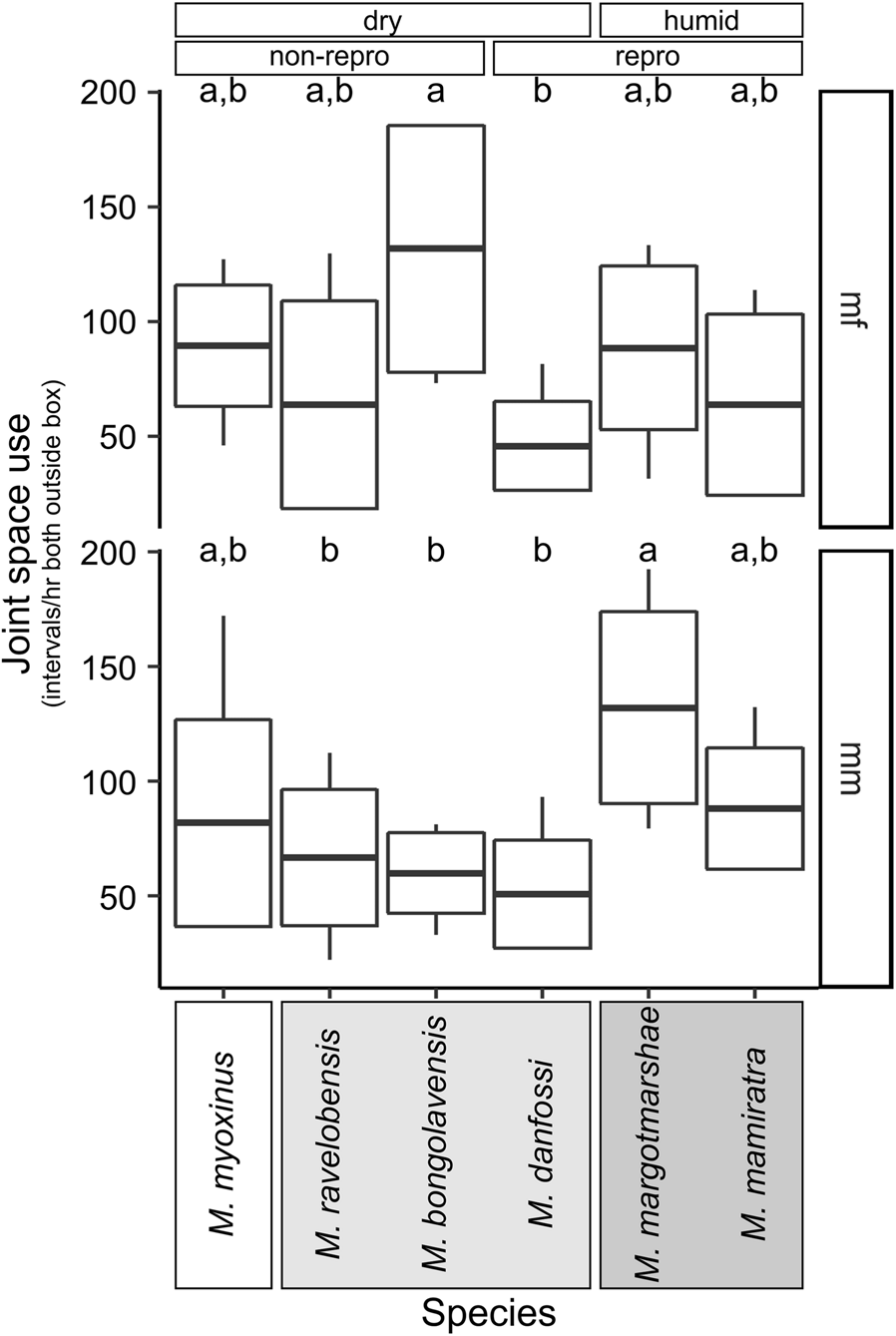
Species comparison of *joint space use* per dyad type (interval frequency/hour both outside box). Mean, box: standard deviation, whiskers: minimum, maximum. Groupings according to forest type (dry, humid) and reproductive activity in the population (non-repro: no female reproductively active; repro: some females reproductively active) are indicated above the graph. Taxonomic clades (clade 3: white; clade 2: light grey; clade 1: dark grey, see Additional file 1) are indicated by boxes behind species names. Different small letters on top of box plots indicate statistical differences (p < 0.05) between species

Pair partners stayed together in the same cage compartment in between 21.8 and 192.2 intervals/h that both partners spent outside the sleeping box. *Joint space use* differed again significantly between species, although in this case only the *species* model (#2) explained significantly more variation than model 0 (Additional file 3, Test 1) and fitted significantly better than the *forest*, *clade*, and *repro* models (#3, #4, #5), respectively (Additional file 3, Test 2). The addition of the variable *pair type* improved the model fit significantly (Additional file 3, #6), suggesting that male–female dyads and male–male-dyads differed in their *joint space use* (Fig. 3). As a result, both datasets (mm-dyads, mf-dyads) were analyzed separately in a second step.

**Fig. 4:**
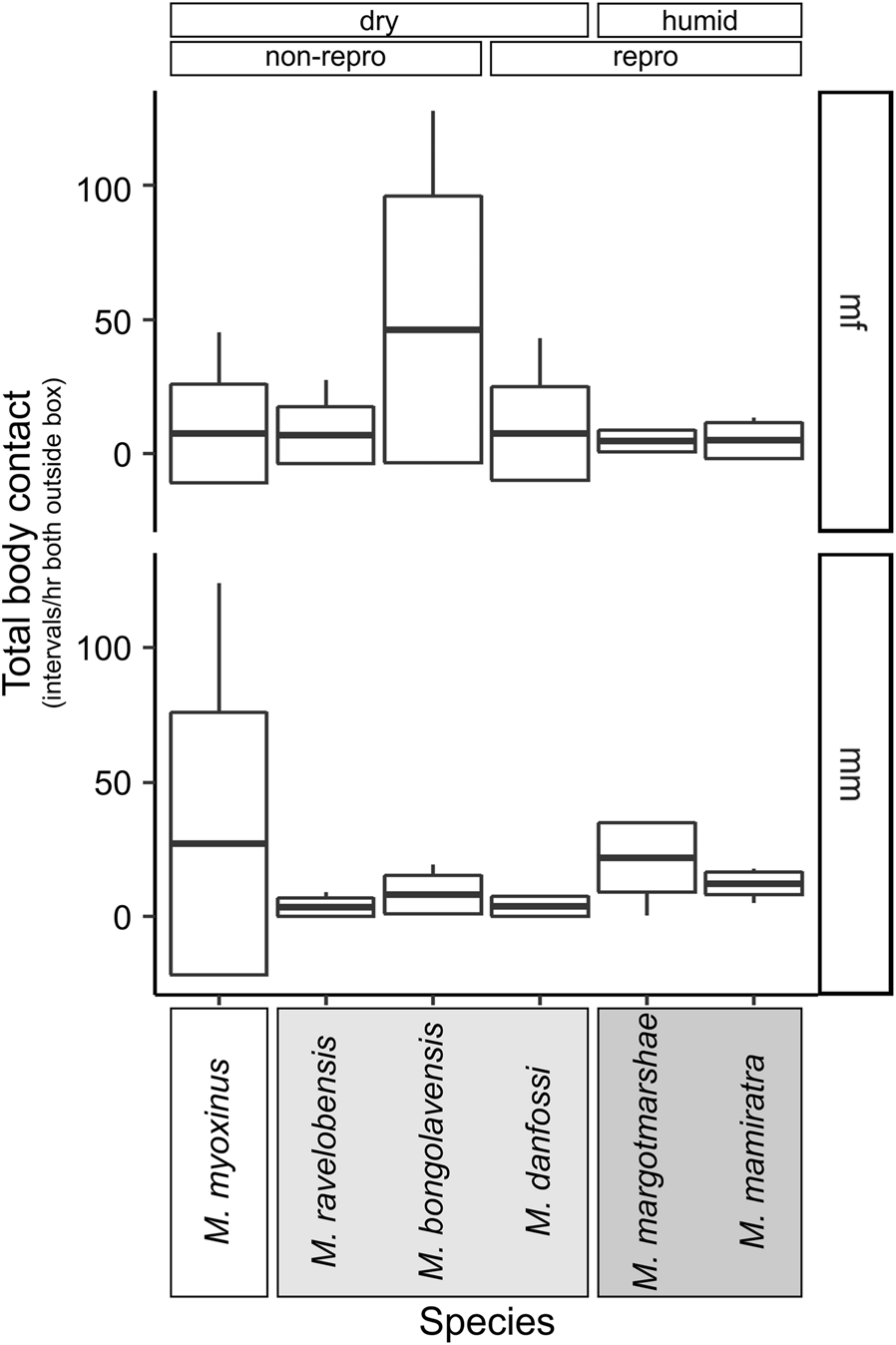
Species comparison of *total body contact* per dyad type (interval frequency/hour both outside box). Mean, box: standard deviation, whiskers: minimum, maximum, Groupings according to forest type (dry, humid) and reproductive activity in the population (non-repro: no female reproductively active; repro: some females reproductively active) are indicated above the graph. Taxonomic clades (clade 3: white; clade 2: light grey; clade 1: dark grey, see Additional file 1) are indicated by boxes behind species names

The *joint space use* of male-female dyads was best explained by the *species* model (#8), which was the only significant model among all (#8–11, Additional file 3, Test 1) and fitted significantly better than the *forest*, *clade*, and *repro* model (#8–11, Additional file 3, Test 2). Male–female dyads of *M. bongolavensis* stayed most frequently together in the same compartment (median = 138.6 intervals/h, min = 73.2, max = 184.6, Fig. 4), which accounted on average for more than half of the intervals (57.8%) that both dyad partners spent outside the box. A posthoc test revealed a significant difference to *M. danfossi* with the smallest median of 38.0 intervals/h (min = 9.6, max = 81.4) which accounted on average for only 15.8% of the intervals outside the box. None of the other comparisons were significant.

The variations in joint space use of male–male dyads could not be best explained with one single model, i.e. the *species*, *forest* or *clade* model (#13–15) had all very similar AIC and BIC values (Additional file 3, Test 1). Qualitatively, mm-dyads of *M. margotmarshae* and *M. mamiratra* showed the highest average rates of joint space use, whereas *M. danfossi* had the lowest rates as in the mf-dyads (Fig. 3). The details of the three models and the posthoc test for the *species* model revealed that (1) *M. margotmarshae* had significantly higher rates of *joint space use* than *M. ravelobensis*, *M. bongolavensis* and *M. danfossi*, (2) species living in humid forest had higher rates than species living in dry forest, and (3) species of the northwestern clade had significantly lower rates of *joint space use* than species from the northern clade (Additional file 3).

The dyads spent between zero and 127.7 intervals per hour outside the sleeping box in physical contact with each other. These variations could not be statistically explained by any single parameter model (Additional file 4, Test 1), neither the *species*, *forest*, *clade* nor the *repro* model. However, when adding *pair type* to the *species* model (#6), it fitted significantly better than the null model (Additional file 4, Test 2, Fig. 4). As in the case of *joint space use*, the total body contacts in mf-dyads could be best explained by the *species* model (Additional file 4, #8), although post hoc tests only revealed two statistical trends for differences between *M. bongolavensis* vs. *M. myoxinus* and *M. margotmarshae* which had lower values, respectively (Fig. 4).

In the case of mm-dyads, the *forest* model (#14) and the *clade* model (#15) explained the variation better than the null model (#12, Additional file 4) and they performed equally well, indicating that (1) mm-dyads from the humid forest had more body contacts than those from the dry forest, and (2) mm-dyads from the northwestern clade had significantly less body contacts than those from the northern clade (Additional file 4, Test 2).

Overall, the results on total body contacts largely correspond to the findings on the joint space use, and both variables indeed correlated significantly with each other (Spearman Rank correlation test, r_S_ = 0.572, n = 71, p < 0.0001). In contrast, neither of these two variables correlated significantly with the joint use of the sleeping box (r_S-joint space use_ = − 0.1032, n = 71, p = 0.3917, r_S-total body contact_ = − 0.1750, n = 71, p = 0.1443).

Co-feeding occurred only rarely (overall median = 1), between zero and 15 times per dyad across the entire observation period, and the species-specific medians varied only slightly between zero times (*M. margotmarshae*, *M. myoxinus*), 0.5 times (*M. danfossi*, *M. ravelobensis*), once (*M. bongolavensis*) and twice (*M. mamiratra*) across the 9–18 h of observations per dyad. Given the rarity of this behavior, this behavior was not submitted to statistical modelling.

### Variation in rates of aggression and number of conflicts among dyad types and species

Individuals showed aggressive behaviors towards their dyad partners on average 2 ± 4.3 times per hour outside the sleeping box and varied between zero and 36.0 times across all individuals. Aggression rates could potentially be influenced by species (*species* model), forest type (*forest* model), the phylogenetic background (*clade* model), the presence of a reproductively active female (*repro* model) in the dyad, but also by the *individual dyad* (random factor), the *sex* of an individual and the *dyad type* (mf or mm). In a first step, the relative suitability of the first four models to explain aggression rates was tested, whereas the *sex* and *dyad type* entered the models only afterwards.

**Fig. 5:**
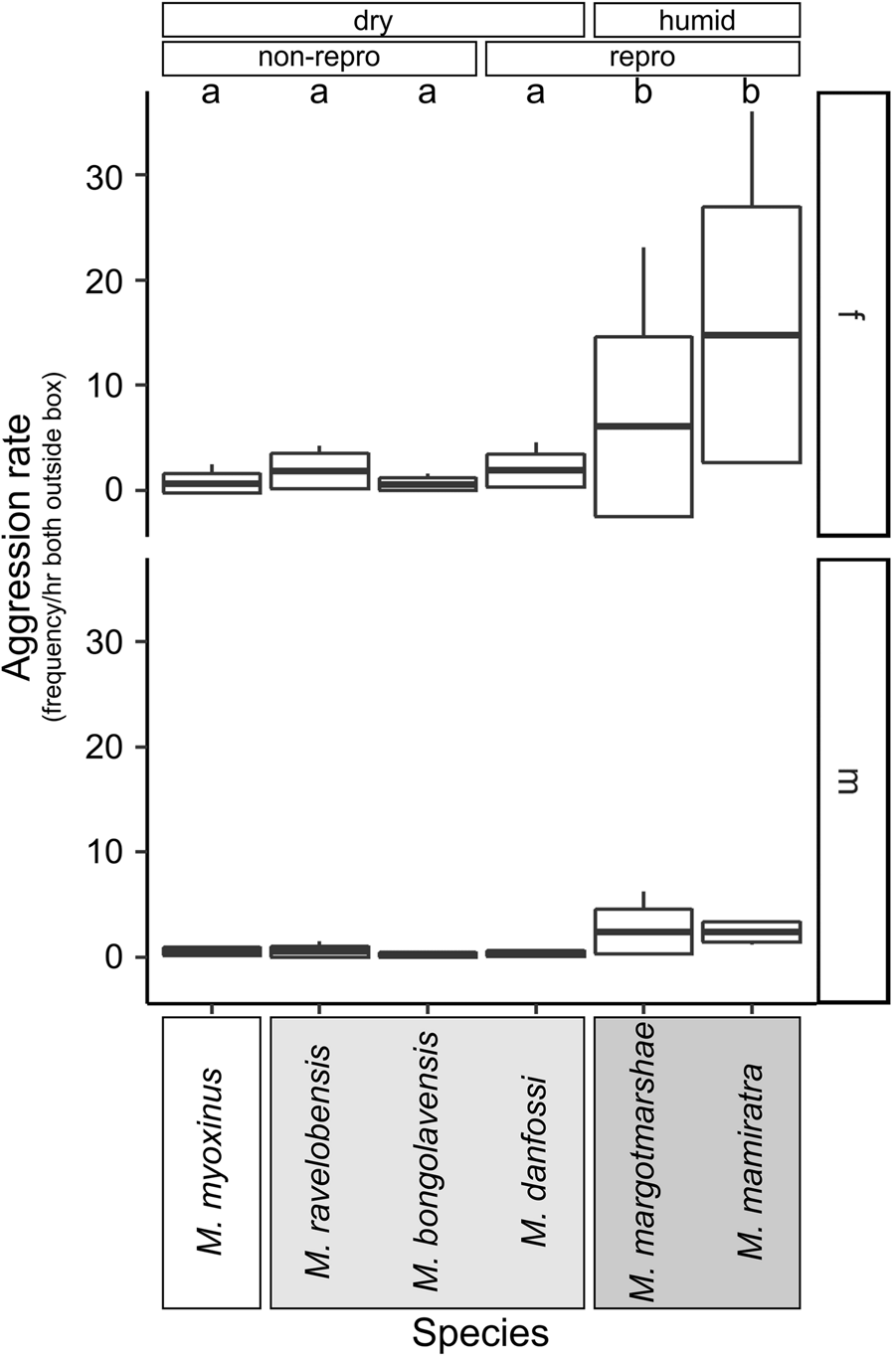
*Aggression rates* in mf-dyads for each species and sex separately (interval frequency/hour both outside box). Mean, box: standard deviation, whiskers: minimum, maximum. Groupings according to forest type (dry, humid) and reproductive activity in the population (non-repro: no female reproductively active; repro: some females reproductively active) are indicated above the graph. Taxonomic clades (clade 3: white; clade 2: light grey; clade 1: dark grey, see Additional file 1) are indicated by boxes behind species names. Different small letters on top of box plots indicate statistical differences (p < 0.05) between species

Three models, the *species*, *forest*, and *clade* model, provided a significantly better fit to the data than the null model (Additional file 5, Test 1, models #2–#4) and performed equally well. In all three models, the addition of the *dyad type* and *sex* improved the fit significantly and a choice between them was not possible at that stage (Additional file 5, Test 2 models #6–#8). However, within mf-dyads, the *species* model fitted the data best when *sex* was included as a further variable (Additional file 5, #10, Fig. 5). A Tukey test revealed that *M. mamiratra* and *M. margotmarshae* had significantly higher aggression rates than all other species, whereas both species differed from each other by a statistical trend (Estimate = 0.5798, SE = 0.2119, z = 2.737, p = 0.0682). Moreover, males had significantly lower aggression rates than females, which was particularly evident in *M. margotmarshae* and *M. mamiratra* (Fig. 5).

In mm-dyads, only the *forest* model (Additional file 5, #15) performed significantly better than the null model with mm-dyads in humid forest showing significantly higher rates of aggression than those living in dry forests (Additional file 5, Fig. 6).

**Fig. 6:**
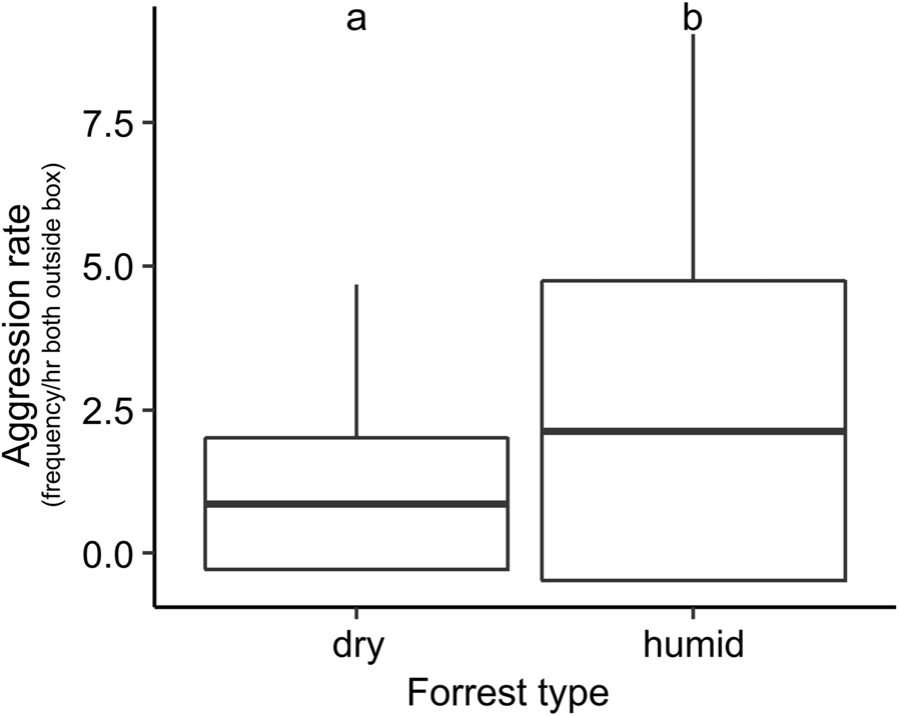
*Aggression rates* in mm-dyads for each forest type (interval frequency/hour both outside box). Mean, box: standard deviation, whiskers: minimum, maximum. Different small letters on top of box plots indicate statistical differences (p < 0.05) between forest types

A total of 2101 conflicts were observed across the entire study period. The number of conflicts between pair partners varied widely between zero and 313 conflicts (mean = 29.6 ± 53.3 SD) per dyad and these were not significantly correlated with the *joint space use* of dyad partners (Spearman Rank correlation test, r_S_ = − 0.1003, n = 71, p = 0.405). All four models (*species*, *forest*, *clade*, *repro*) performed significantly better than the Null model, but the *species* model fitted the data best (Additional file 6, #2). The interaction of species*pair type improved the model significantly (#6) and both dyad types were therefore modelled separately (Additional file 6).

The *species* model (#8) performed best among all four models (#8–#11) for the mf-dyads (Additional file 6, Test 2) and revealed that mf-dyads of *M. mamiratra* had significantly more conflicts than those of any other species (Additional file 6, Fig. 7). No other significant difference was detected. The number of conflicts in mm-dyads could not be explained by one model alone, but the *species* model (#13), the *forest* model (#14) and the *clade* model (#15) all showed a significant fit to the data, indicating that mm-dyads in (1) *M. mamiratra* had significantly higher conflict numbers than *M. myoxinus*, (2) humid forests had higher conflict numbers than those in dry forests, and that (3) mm-dyads from the western and northwestern clade had significantly lower conflict numbers than those in the northern clade.

**Fig. 7:**
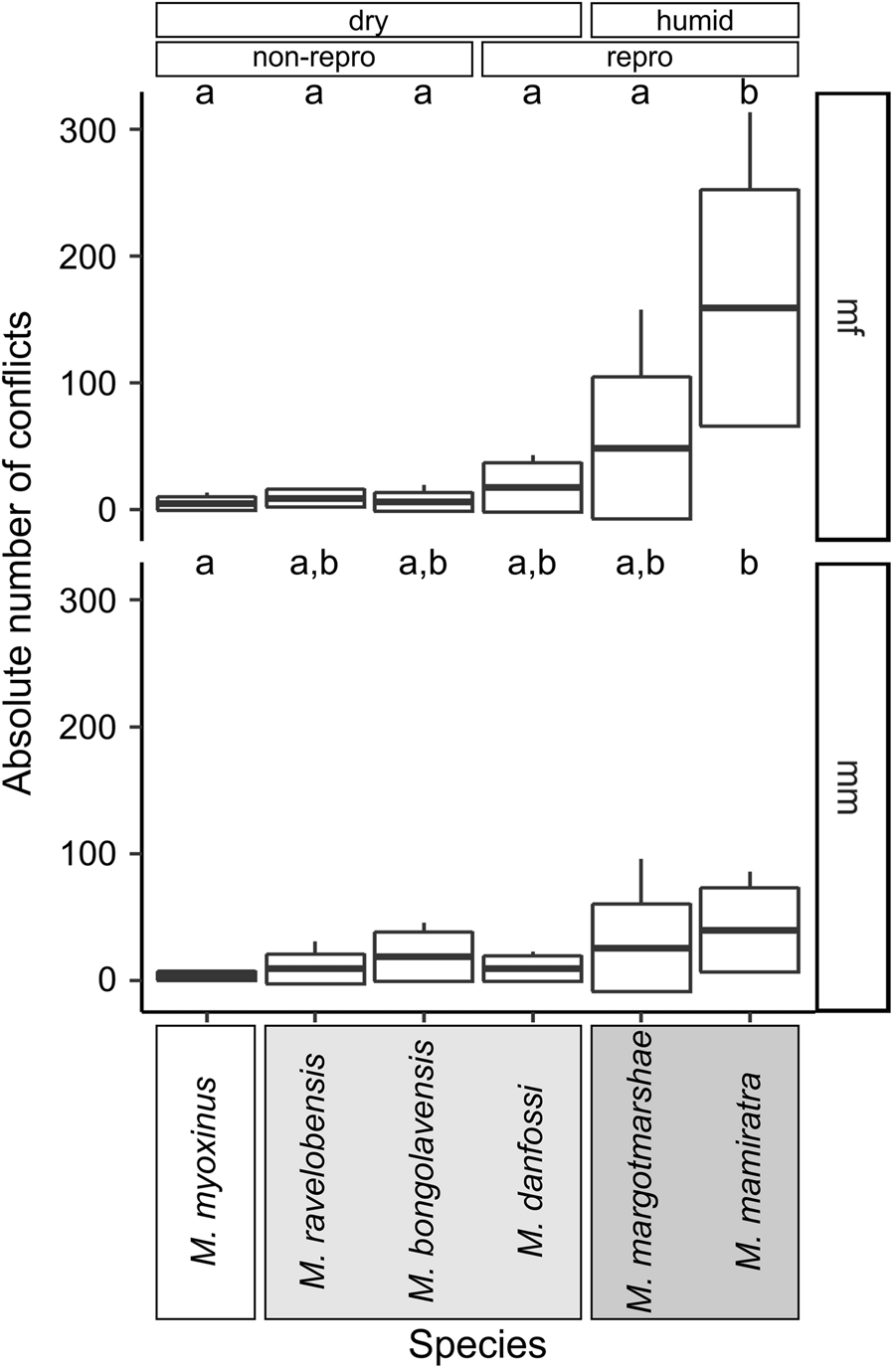
Species comparison of the *total number of conflicts* per dyad type. Mean, box: standard deviation, whiskers: minimum, maximum. Groupings according to forest type (dry, humid) and reproductive activity in the population (non-repro: no female reproductively active; repro: some females reproductively active) are indicated above the graph. Taxonomic clades (clade 3: white; clade 2: light grey; clade 1: dark grey, see Additional file 1) are indicated by boxes behind species names. Different small letters on top of box plots indicate statistical differences (p < 0.05) between species

### Variation in social dominance among dyad types and species

The total number of conflicts won by each dyad partner was used to determine whether social dominance could be statistically confirmed within each dyad (Additional file 6). Intersexual dominance was detected in 16 of 36 mf-dyads (44.4%) but these were not evenly distributed between species (Table 1). Species ranged from no female dominance (*M. myoxinus*) via rare female dominance (*M. ravelobensis*, *M. bongolavensis*), and moderate female dominance (i.e. half of the dyads showing female dominance, *M. danfossi* and *M. margotmarshae*) to unambiguous female dominance (*M. mamiratra*) where all females were dominant over their male partners. In contrast, only one case of male dominance could be detected (*M. margotmarshae*). Across all six dyads, females won significantly more conflicts than males in two species only, *M. danfossi* and *M. mamiratra* (Table 1). Female dominance did not depend on body mass differences, as dominant females were partly lighter (n = 6), heavier (n = 7) or equal in body mass (n = 2) to their male dyad partners (Additional file 7).

**Table 1:** Number of won conflicts for males and females in male–female dyads and their statistical comparison

Species	N	Median		Quartiles		Min–max		Wilcoxon test		Dominance	
		m	f	m	f	m	f	Z	*p*	m	f
*M. myoxinus*	6	0.5	1	0–1	0–1	0–2	0–3	0.404	0.686	0	0
*M. ravelobensis*	6	0.5	2	0–2	0–9	0–2	0–14	1.604	0.109	0	2
*M. bongolavensis*	6	0	2	0	0–4	0–4	0–8	1.095	0.273	0	1
*M. danfossi*	6	0.5	8	0–3	1–25	0–4	1–41	*2.201*	*0.028*	0	3
*M. margotmarshae*	6	1.5	13.5	0–9	2–25	0–16	1–142	1.572	0.116	1	3
*M. mamiratra*	6	4	101	3–5	48–196	1–10	46–298	*2.201*	*0.028*	0	6

N: total number of dyads; min–max: minimum to maximum value; m: male; f: female; Italic: p < 0.05; dominance: number of mf-dyads with male or female dominance

Statistical values are in italics

Male–male dominance was detected only in four out of six species and within these in 13 of 23 dyads (56.5%, Table 2). Species varied between 0% dyads with male dominance (*M. myoxinus*, *M. ravelobensis*) to 80% dyads with male dominance (*M. bongolavensis*). The number of significant dominance relationships in mf-dyads and mm-dyads per species did not correlate with each other (Spearman Rank correlation test, r_s_ = 0.478, n = 6, n.s).

**Table 2:** Number of male–male-dyads with significant male–male dominance per species

Species	N	Dominance
*M. myoxinus*	6	0
*M. ravelobensis*	6	0
*M. bongolavensis*	5	4
*M. danfossi*	6	2
*M. margotmarshae*	6	3
*M. mamiratra*	6	4

N: total number of dyads; dominance: number of dyads with male dominance

## Discussion

The aim of this study was to analyze and compare the patterns of social tolerance (inferred by non-agonistic contacts and proximity) and its consequences (inferred by aggression rates, number of conflicts and social dominance) in a potentially competitive situation between six species of mouse lemurs that belong to different phylogenetic clades, inhabit different forest types and were studied in different reproductive periods. For a solid interpretation of the results, however, it is first essential to evaluate, if all proximity parameters indeed reflect social tolerance between dyad partners. This is important, since the animals were most likely not familiar with each other and were kept together in temporary confinement in a cage setting. Under these circumstances, putative signals of social tolerance such as the *joint stay in sleeping box* or the joint stay in one cage compartment (*joint space use*) could also reflect a crypsis response [50] and point towards a lack of habituation [51]. This question was addressed by correlating the proximity parameters (*joint stay in sleeping box*, *joint space use*) with an intrinsically meaningful parameter, the interval frequency of non-agonistic body contacts. This analysis revealed that the *joint stay in the sleeping box* is most likely not reflecting true affiliation between the dyad partners, since there was no correlation detected with the total number of non-agonistic body contacts. The *joint stay in the sleeping box* was highest in *M. myoxinus* (Fig. 2), although this species showed rather low frequencies of non-agonistic body contact (Fig. 4). We therefore conclude that the joint stay in the sleeping box is reflecting some degree of disturbance that was experienced by the dyads during observations. Whereas the conditions for the behavioral observations themselves were always identical (e.g., distance between observer and cage, light regime, cage dimensions and furbishing), this was not the case for the external conditions. When working with *M. myoxinus*, the cages were placed under trees not far from the next village (for safety reasons) with a path leading close by that was frequented by villagers even at nighttime. It is likely that this external source of disturbance may have negatively influenced the behavior of the animals in the cages, i.e. they were hiding longer in the sleeping boxes than the individuals of the other species. As a result, this parameter will not be included in the subsequent discussion. In contrast, there was a positive correlation between *total body contacts* and the *joint space use* (p < 0.0001), but no correlation between *joint space use* and the *number of conflicts* (n.s.). This parameter can thus be confirmed to be useful for the description of social tolerance between individuals.

### Influence of phylogeny on social tolerance

Phylogenetic relatedness has previously been shown to influence and constrain a wide variety of behavioral patterns in primates ranging for example from feeding habits [52], patterns of reproduction [36], cognitive function [53, 54], communication [55–57], dominance styles [58, 59], to infant rearing systems [60, 61]. We therefore hypothesized that the social tolerance patterns of the six studied mouse lemur species may reflect their membership in three phylogenetic clades that were well supported in several previous phylogenetic studies [28, 62–64]. However, there was only relatively weak support for phylogenetic effects on social tolerance and its consequences in mouse lemurs (Table 3). The models revealed significant support for the *clade* model only for three variables (j*oint space use*, *total body contact*, *number of conflicts*) and only for male–male dyads. Furthermore, in all three cases, the support was not exclusive for the *clade* model, but significant support also existed for the *forest* model and/or the *species* model.

**Table 3:** Summary of findings on parameters that explained variation in social tolerance and its consequences

Parameter	Pair type	Clade	Forest	Repro	Species	Directionality
**Social tolerance**						
Joint stay in sleeping box	Both	–	–	–	X	*Mmyo *> *Mbon*, *Mmar, Mmam* *Mmam *< *Mdan*, *Mrav*
Joint space use + variable *pair type*	mf	–	–	–	X	M*bon *> *Mdan*
	mm	X	X	–	X	*Mmar *> *Mrav*, *Mbon*, *Mdan* HF > DF N > NW
Total body contact + variable *pair type*	mf	–	–	–	X	*Mbon* > *Mmyo*, *Mdan*
	mm	X	X	–	–	HF > DF N > NW
**Consequences**						
Aggression rate + variable *pair type*	mf	–	–	–	X	*Mmam, Mmar *>* Mmyo*, *Mrav*, *Mbon*, *Mdan* M < F
	mm	–	X	–	–	HF > DF
No. of conflicts + variable *pair type*	mf	–	–	–	X	*Mmam *>* Mmar*, *Mdan*, *Mbon*, *Mrav*, *Mmyo*
	mm	X	X	–	X	*Mmam *>* Mmyo* HF > DF N > NW, W
Intersexual dominance	mf	–	–	x	x	*Mmam *>* Mmar*, *Mdan* > rest (qualitatively)
mm-dominance	mm	–	–	–	x	*Mmam*, *Mbon *>* Mmar*, *Mdan* > rest (qualitatively)

*Mmyo: M. myoxinus*; *Mrav: M. ravelobensis*; *Mbon: M. bongolavensis*; *Mdan: M. danfossi*; *Mmar*: *M. margotmarshae*; *Mmam: M. mamiratra*; mm: male–male dyads; mf: male–female dyads; X: statistical support from mixed models; x: qualitative support from tabulated data; HF: humid forest; DF: dry forest; N: northern clade; NW: northwestern clade; W: western clade

Concerning the agonistic behavior (aggression rates, no. of conflicts, Figs. 5, 7), the four species of the northwestern and western clade (*M. myoxinus*, *M. ravelobensis*, *M. bongolavensis*, *M. danfossi*) showed rather similar patterns, but typically differed from one or both members of the northern clade (*M. mamiratra*, *M. margotmarshae*). However, members of the same clade were not always similar in their behavior. For example, male–female dyads of *M. bongolavensis* showed significantly more *joint space use* and *total body contacts* than *M. danfossi*, and the *number of conflicts* was significantly higher in mf-dyads of *M. mamiratra* than of *M. margotmarshae*.

### Influence of habitat type on social tolerance

Based on temperature and rainfall data [36, 65], the warm lowland evergreen rainforests inhabited by *M. mamiratra* and *M. margotmarshae* at the northern end of the study region are the least seasonal habitats and contrasted largely with the dry deciduous forests where the other four mouse lemur species occurred. It was hypothesized that species inhabiting the dry forests should be energetically more constrained by seasonal food scarcity during the dry season than those living in humid forests [66]. As a consequence, species in the resource-poor dry forests should be more competitive when confronted with a monopolizable resource (one clumped feeding bowl with banana) and show higher levels of aggression/conflicts and lower levels of social tolerance than species inhabiting the resource-rich humid forest.

The statistical tests revealed ambiguous support for this hypothesis (Table 3). Forest models were statistically supported only in male–male dyads in most parameters, but the effect was not always as expected. Whereas male–male dyads in humid forests showed indeed higher social tolerance (*joint space use*, *total body contact*) than those in the dry forest (in line with the expectation), they also had higher aggression rates and more agonistic conflicts than those in the dry forest (in contrast to the expectation).

Male–female dyads in humid vs. dry forest did not differ significantly neither in social tolerance nor in its consequences (Table 3). Whereas the mf-dyads of *M. bongolavensis* (dry forest) showed the highest average level of social tolerance (*joint space use* and *total body contact*) of all species, *aggression rates* and *number of conflicts* were highest in *M. mamiratra* followed by *M. margotmarshae* (both humid forest). Based on this rather small dataset, these findings cannot be well interpreted. Further data would be desirable, for example on energetic constraints, feeding regimes and the extent of resource competition within each of these species, to fully understand the basis for these observations.

### Influence of reproductive activity on social tolerance

It is known from the gray mouse lemur (*M. murinus*) that males can compete severely for the access to estrous females under natural conditions (*M. murinus*, [15]) and in captivity (*M. murinus*, [67]), and that conflict rates among the sexes are higher and female dominance is more expressed in the reproductive season than in the non-reproductive season, at least in captivity (*M. murinus*, *M. lehilahytsara*, [42]). It was therefore predicted that social tolerance (i.e. *joint space use*, *total body contact*) should be lower and aggression rates and conflict numbers should be higher when females are reproductively active in the population or in the dyad. It needs to be stated, though, that the period of reproductive activity of the six study species was not known prior to this study and was only established in parallel [36]. In retrospective, this prediction implies that three species, *M. mamiratra*, *M. margotmarshae* and *M. danfossi*, should have shown similarly low levels of social tolerance, higher *aggression rates* and *number of conflicts*, and should have contained more dyads with clear dominance relationships and female dominance than the other species that did not contain reproductively active females (Additional file 1).

However, the only detected similarity between the three species was that they contained the highest number of female dominant dyads (FDD) with either 50% FDD (*M. margotmarshae*, *M. danfossi*) or even 100% FDD (*M. mamiratra*) (Table 1) in contrast to the other three non-reproductive species that ranged between 0% FDD (*M. myoxinus*) to 33% FDD (*M. ravelobensis*). Besides, no single statistical model was in support of the predicted patterns (Table 3), although small sample sizes preclude a final statement. Instead, *M. danfossi* showed rather similar results in almost all analyses to the other mouse lemur species living in the dry forests, whereas *M. mamiratra* and *M. margotmarshae* showed intermediate tolerance levels and high *aggression rates* in both dyad types. Future studies will be needed on the socioecology of these species to clarify the reasons for these discrepancies.

### Interspecific variability in social tolerance and implications for social diversity in mouse lemurs

The comparative evaluation of the *clade* model, *forest* model and *repro* model against a simple *species* model revealed that in male–female dyads, the variation in all parameters could be best explained by species differences. This overall support for the simple *species* model is the consequence of single species acting as outliers in the analyses (Table 3). This was the case in the *joint space use* (*M. bongolavensis* high, *M. danfossi* low), *total body contact* (*M. bongolavensis* high), *aggression rates* (*M. mamiratra* and *M. margotmarshae* high), *number of conflicts* (*M. mamiratra* high), female dominance (*M. mamiratra* high), and in male–male dominance (*M. mamiratra* and *M. bongolavensis* high). Given this distribution, two species, *M. bongolavensis* and *M. mamiratra*, require a separate discussion.

**Fig. 8:**
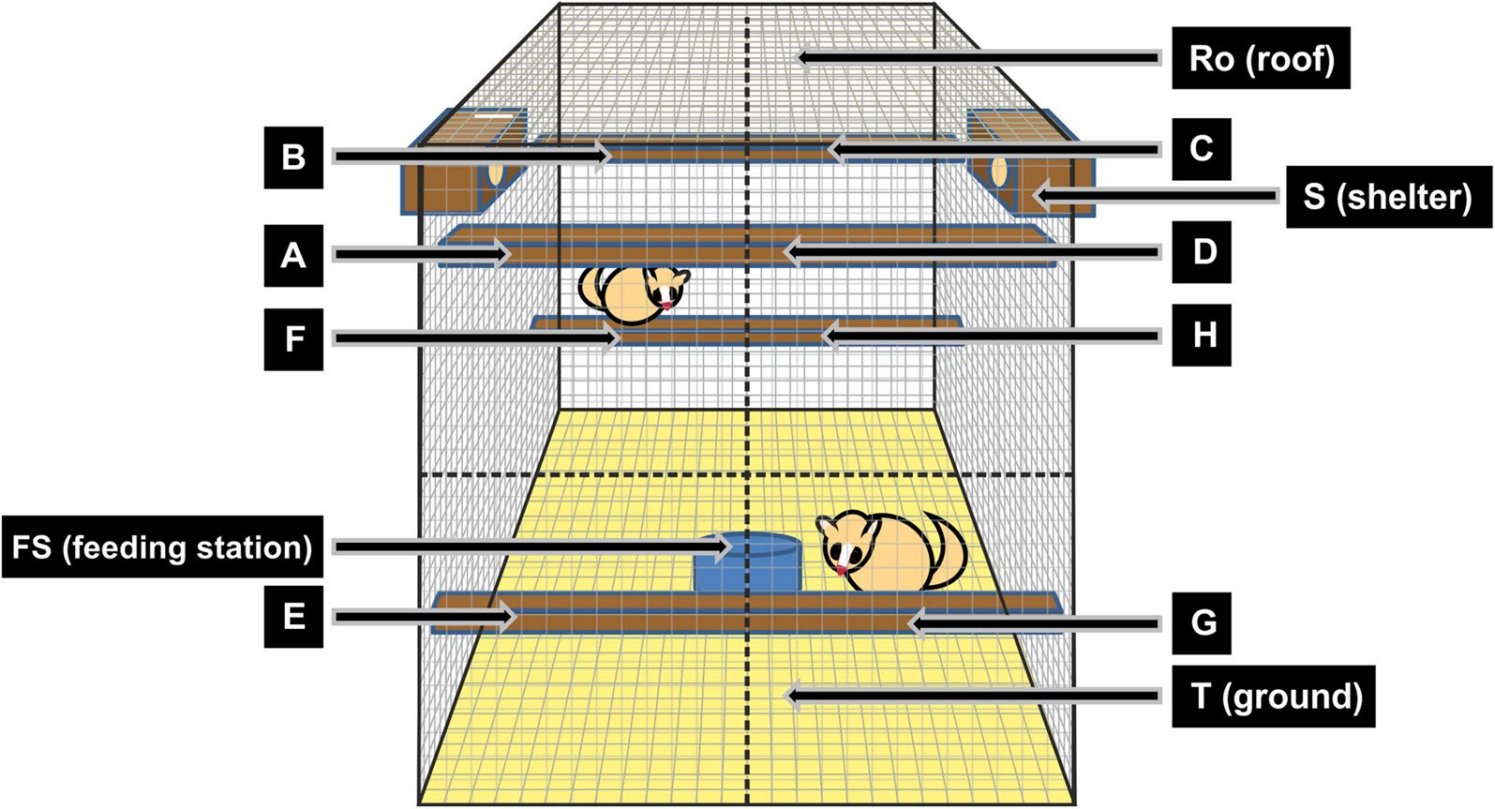
Encounter cage with eight compartments (A–H) and four extra locations (Ro, T, S, FS). One sleeping box (S) was attached per side. Food was presented on the cage floor in a bowl (FS). Upper and lower front and back compartments were equipped with one wooden bar each

Male–female dyads of *M. bongolavensis* were characterized by strikingly high social tolerance, i.e. dyad partners stayed on average more than half of the intervals (57.8%) together in the same cage compartment and also had the highest average values of *total body contacts* of all species. It is known that the reproductive period of *M. bongolavensis* starts towards the end of August (Radespiel, Rakotondravony unpubl. data) and none of the trapped females was showing signs of estrous in the current study that finished around Mid-August (9th July–13th August). It is, however, possible that the males were already quite interested in the females and approached them frequently and maintained proximity during the observations. It is known from gray mouse lemurs that males start actively searching in an enlarged home range for estrous females even 1 month before the onset of the reproductive season [68], whereas females do not change their use of space, but announce their receptivity by vocal and olfactoric signalling [69]. Unfortunately, no data are available on the frequency of approaches by males versus females of *M. bongolavensis* in the cage experiments, so that it cannot be decided, which sex was responsible for the proximity between them. Since this elevated proximity was not accompanied by elevated aggression rates or conflict numbers (Figs. 5, 7 and Table 1), though, it can be assumed that the females at least tolerated the males, which is unusual, at least in gray mouse lemurs [38, 70]. Interestingly, male–male dyads of *M. bongolavensis* had among the highest number of clear dominance relationships (n = 4), and their conflict numbers were also slightly elevated (Fig. 8). These findings suggest increased levels of intrasexual competition among males prior to the onset of mating activities, which has already been described from the gray mouse lemur [67]. Interestingly, although *M. myoxinus* was also studied 1 month prior to the onset of estrus (study period: 9th September–13th October; estrus may start in October, [36], it did not show the same behavioral pattern as *M. bongolavensis*. This suggests that social tolerance and its consequences are multi-facetted in mouse lemurs and not easily explained by single parameters. Other factors, such as age, experience [e.g., 25, 42] or consistent personality differences [71] may also have influenced the behavior of dyad partners during these encounters but could not be investigated with this dataset.

Mf-dyads of *M. mamiratra* were different from the other species in various ways. They were very active (rare use of sleeping box), had intermediate levels of social tolerance, showed very high female *aggression rates*, high *conflict numbers* and unambiguous female dominance, i.e. all females won significantly more conflicts than their male partners. The difference between the number of conflicts won by males (median = 4, min = 0, max = 10) and by females (median = 101, min = 46, max = 298) was strikingly larger than in any of the other species (Table 1). As the observed *M. mamiratra* females were in different reproductive states (3× no estrous, 1× pro-estrous, 1× recently in estrous, 1× pregnant), these consistently high values cannot be explained with a specific reproductive state (Additional file 1). In addition, four out of six male–male dyads had significant male–male dominance relationships, indicating elevated levels of male intrasexual competition even when not being in contact with females (Table 3, Fig. 7). There are no socioecological data available on this species. A recent study suggested that they, together with *M. margotmarshae*, may be exceptional within mouse lemurs as they may have a less seasonal reproduction than the other species studied so far [36]. A release from seasonality may increase the monopolization potential and therefore could result in higher contest competition between males [15]. However, the costs and benefits of sociality in males and females, the underlying competitive regimes and the proximate and ultimate reasons for their very strong female dominance cannot be inferred for this species from these first datasets.

Finally, one interesting result of this study is the variable expression of female dominance among and within the six study species. Whereas intraspecific variations may be proximately explained by differences in age, experience, reproductive state or personality [25, 42, 71], interspecific differences are still subject of debate. Previous studies have suggested that this variability may be ultimately explained by variations in sex-specific energetic constraints that the species underwent in their ancestral habitats and by differences in the species-specific social organization and social structure [24, 25]. However, to evaluate the differences between the species in more depth, it would be important to compare dominance data that were all collected during the same reproductive period [25], i.e. either within or outside the period of reproduction.

## Conclusions

This study revealed substantial evidence for variation in social tolerance (i.e. the patterns of affiliation) and its consequences (i.e. aggression rates, number of conflicts and social dominance) in a potentially competitive situation in mouse lemurs, one of the most speciose radiation of lemurs in Madagascar. Whereas some variation can be explained by ecological factors, other species-specific differences cannot be easily understood. For example, social tolerance levels differed between *M. bongolavensis* and *M. myoxinus*, although both were studied within the same reproductive period and in the same forest type, and *M. mamiratra* and *M. margotmarshae* differed in aggression and female dominance, despite inhabiting the same forest type and containing reproductively active females. These findings suggest that the regulation of social tolerance and its consequences is much more complex than previously thought and that mouse lemurs show strong signals of behavioral plasticity. Several authors have criticized over the last decade the increasing number of lemur species that have been scientifically described based mainly on genetic results (e.g., [72–74]). Results like the ones presented here suggest, however, that the different taxa also behave differently in a standardized social encounter paradigm. Under the concept of integrative taxonomy [75, 76], these findings can therefore be interpreted as support for the taxonomic distinctiveness of these lineages.

## Methods

### Study sites and study species

This study was conducted at six sites (Bombetoka, Ampijoroa, Marosely, Anjiamangirana, Ankaramibe and Lokobe) situated in six Inter-River-Systems (IRS 0, I, II, III, V and IRS VI) [77] from western to northern Madagascar (Fig. 1, Additional file 1). All six species are threatened by habitat loss in their natural habitats and are therefore classified as endangered (EN, *Microcebus myoxinus*, *M. ravelobensis*, *M. bongolavensis*, *M. danfossi*, *M. margotmarshae)* or critically endangered (CR, *M. mamiratra*) according to the IUCN red list [78]. The study species differ in forest type (resource-poor dry deciduous forest vs. resource-rich evergreen humid forest), their clade membership (clades 1, 2, 3), and the presence or absence of reproductively active females in the population (Additional file 1). For logistic reasons (not all field sites are accessible throughout the whole year) all field work took place during the two successive dry seasons from May to October in 2015 and in 2016.

### Capture and selection of study animals

At all dry forest sites, *Microcebus* spp. were captured with Sherman live traps (HB Sherman Traps Inc., Tallahassee, FL), baited with banana slices according to established methods [36, 77, 79], whereas they were captured by hand in the two humid forest sites, as mouse lemurs are not attracted to traps in resource-rich humid habitats. Capture by hand was performed in rather small and somewhat isolated trees or bushes at nighttime. Hand-captured animals are directly transferred to a cloth bag or Sherman trap and calmed down quickly, probably since such a small dark place corresponds well to their natural sheltered sleeping sites [50]. They were then transferred to camp for routine handling, during which they were sexed, weighed and marked according to established protocols [77, 79].

Dyad partners for social encounter experiments were selected based on comparable body mass and on being trapped as far away as possible from each other (mf-dyads: median = 244 m, quartiles = 128–1145 m, min = 25 m, max = 2406 m, N = 36; mm-dyads: median = 350 m, quartiles = 88–1140 m, min = 0 m, max = 2549 m, N = 35) to reduce the chances for prior familiarity between dyad partners. For individual identification during the nocturnal observations, one animal of each dyad was marked with a fur cut on the tail. Animals not selected for social encounter experiments were released at their trapping site in the early evening. As a rule, a total of six male–male and six male–female dyads were formed per species with each individual being included in only one dyad. Each dyad was observed for a maximum of six nights following the night of capture. As an exception and due to capture problems, we formed only five male–male dyads in *M. bongolavensis*. All animals were released after 1 week at their individual capture point after the end of the last night of observations. Such a temporary stay of wild mouse lemurs in cages has been employed in several previous experimental studies and adverse effects were not observed neither on the individual nor on the population level (e.g., [24, 51, 80–83]).

### Experimental set-up and data collection

The social encounter experiments were conducted in cages of about 1 m^3^ that were equipped with four wooden bars and two sleeping sites (Fig. 8, [80]). Cages were placed in the forest close to the research camp but > 1 km away from the capture sites. Animals were fed daily with banana and received water ad libitum. Furthermore, they fed on arthropod prey that entered their cages naturally during the night. Banana is a highly preferred food of mouse lemurs and the well-being of all animals was checked daily. No health issues occurred during the experiments.

Only one shelter was available for the dyad partners during observations, while both were accessable outside those times. This approach was chosen to promote social interactions between the partners. Each cage was divided into eight compartments of equal size [upper front (A, D) upper back (B, C), lower front (E, G), lower back (F, H)] in addition to the roof, floor, shelter, and feeding station.

### Behavioral observations

Observations were performed on each dyad during 3 h per night over six consecutive nights between 06h00 p.m. and 09h00 p.m. in 67 of the 71 dyads (94.4%) of the dyads. Two mm-dyads of *M. myoxinus* and one mf-dyad of *M. ravelobensis* were only observed across five nights, and one mm-dyad of *M. myoxinus* was only observed across three nights. Observations ended earlier than planned in these cases due to an unintentional escape of the animals from their cages. During observations, a team of two observers sat motionless in 2 m distance to the cage and started a protocol when the animals woke up. The observers utilized a headlamp and Maglite torch with red filter to obtain better visibility. Protocols were recorded on a digital Dictaphone (Sony).

The use of the 12 locations in the cage was noted every 15 s for both pair partners by means of instantaneous sampling [84]. All occurrences of feeding at the feeding station and of social behaviors of both partners were noted whenever they occurred. Social behaviors consisted (1) of affiliative behaviors (unspecific body contact, allogrooming) that were added up for the purpose of this study to “total body contact”, and (b) of agonistic behaviors (aggressive (A): fighting, chasing, biting, displacing; submissive (S): fleeing, avoidance, for definitions see [85]).

A conflict was defined as a series of agonistic behaviors that was not interrupted for more than one second [85]. A conflict was defined as decided, if (a) one animal behaved aggressively (e.g., chase, displace) and the other one reacted submissively (e.g., fleeing) (AS), (b) one animal avoided the other (OS), or (c) if a physical fight ended in a flight of one opponent (AAS). Social dominance was stated if one individual won significantly more decided conflicts than its partner. It was determined in each dyad separately by means of a Binomial test (http://www.socscistatistics.com/tests/binomial/Default2.aspx), and the overall evidence for intersexual dominance was analysed for each species by means of a Wilcoxon Matched Pairs Test conducted in Statistica 6.0 (StatSoft Inc., Tulsa, OK).

For the purpose of this study, social tolerance was defined as the willingness of individuals to interact non-agonistically with each other and to spend time in proximity to their social partner. On the basis of this operational definition, we inferred social tolerance between the dyad partners by the following behavioral parameters:

- *Joint stay in sleeping box* Number of 15 s-intervals being together in the sleeping box per hour of observations.
- *Joint space use* Number of 15 s-intervals of staying together in one of the eight compartments or on roof or floor of the cage per hour that both animals spent together outside the sleeping box.
- *Total body contact* Number of 15 s-intervals in non-agonistic body contact outside the sleeping box per hour that both animals spent together outside the sleeping box.
- *Co*-*feeding* number of times that both partners were eating together at the feeding bowl.

According to the second approach that is often employed to study social tolerance [3], we also investigated the consequences of social tolerance between the dyad partners in a potentially competitive situation by quantifying the following behavioral parameters:

e. *Aggression rate* Number of individual aggressive behaviors per hour that both animals spent together outside the sleeping box.
f. *Number of conflicts* (see above for definition of conflict).
g. *Social dominance* (see above for definition of dominance).

### Statistical modelling

All spoken protocols were transferred to and edited in EXCEL 2010. Dependent variables were first tested regarding their departure from normal distribution and homogeneity of variances. If needed, data were transformed, either logarithmically (*total body contact*, *aggression rate*) or by square root transformation (*joint space use*, *no. of conflicts*).

Determinants of variation in the *joint stay in sleeping box*, *joint space use*, *total body contacts* and the *number of conflicts* were inferred by means of comparative generalized linear models that were fitted with the *gls*-function and the use of maximum likelihood for the estimation of fixed parameters in RStudio 1.0.143 [86] with the package *nlme*. Each modelling procedure started with a null model (= no fixed factors) and a basic model that included only *species* as fixed factor. Model improvements were tested by means of the *anova()* function and the implemented Likelihood Ratio Test. Next, three alternative models were built with *Clade* (clade 1, 2, 3), *Forest type* (dry vs. humid) and *reproduction* of females in population (yes vs. no) as fixed factors, respectively. The relative improvement provided by these three models over the null model and the *species* model was compared by means of the *anova()* function and the best model was identified by the smallest AIC value given the results of the Likelihood Ratio test. Next, we added the *dyad type* (mm vs. mf) as an interaction term to the best model to test for an improvement of the model fit. If the model was improved significantly, separate models were then fitted to two subsets of the data representing the two dyad types, respectively. Whenever more than two elements were included in one significant factor such as in *species* (six elements = species) and *clade* (three elements = clades), a posthoc test was conducted (Tukey) to identify which species or clades differed significantly from each other.

In order to infer the determinants of the *aggression rate* of individuals, a mixed-effect model was built with the *lme*-function and the use of maximum likelihood for the estimation of fixed parameters in RStudio 1.0.143. *Pair identity* was introduced as random factor, since the behavior of both partners could influence each other. Modelling steps followed those described above with two modifications. First, we used the reproductive status of the paired female as potential determinant of aggression rate of both pair partners (*repro*) instead of the presence of reproductively active female in the capture population. Second, when *dyad type* contributed significantly to model improvement, *sex* (male, female) was introduced as an additional fixed factor to test whether the sexes differed systematically in their aggression rates in mf-dyads.

## Additional files

**Additional file 1.** Species, study site, forest type, latitude (South) and longitude (East) in decimal degree, clade, total number of individual males (M) and females (F) captured at each site, number of studied male–male (MM) and male–female (MF) dyads, study period and number of reproductively active females in observed dyads and in capture population.

**Additional file 2.** Statistical model comparisons and best model to explain the frequency of staying together in the sleeping box (SB) by the parameters species, phylogeny (clade), forest type (forest) or the presence of reproductive females (repro). First, all models were compared to Base 0 model (Test 1, LRT_1_, P_1_-values). Second, the three alternative models were compared to the species model (Test 2, LRT_2_, P_2_-value). Finally, *pair type* was added to the best model (#2) as an interaction term, but did not improve model fit. Model details for the best model are provided below. The best model is highlighted in bold and effect directions are included.

**Additional file 3.** Statistical model comparisons and details of best models to explain the frequency of *joint space use* by the parameters *species*, phylogeny (*clade*), forest type (*forest*) or the presence of reproductive females (*repro*). First, all models were compared to Base 0 model (Test 1, LRT_1_, P_1_-values), Second, the three alternative models were compared to the *species* model (Test 2, LRT_2_, P_2_-value). *Pair type* was added to the best model (#2) as an interaction term and improved the model significantly. Separate models were calculated and compared for mf-dyads and mm-dyads. Model details of the best models are provided. The best model and significant effects are highlighted in bold and effect directions are included.

**Additional file 4.** Statistical model comparisons and details of best models to explain the frequency of *total body contact* by the parameters *species*, phylogeny (*clade*), forest type (*forest*) or the presence of reproductive females (*repro*). First, all models were compared to Base 0 model (Test 1, LRT_1_, P_1_-values), *Pair type* was added to the species model (#2) as an interaction term and improved the model significantly. Separate models were calculated and compared for mf-dyads and mm-dyads. Model details of the best models are provided. Best models and significant effects are highlighted in bold and effect directions are included.

**Additional file 5.** Statistical model comparisons and details of best models to explain *aggression rate* by the parameters *species*, phylogeny (*clade*), forest type (*forest*) or the presence of reproductively active females (*repro*). First, all models were compared to Base 0 model (Test 1, LRT_1_, P_1_-values), *Pair type* and *sex* were added to the models #2–#4 and improved them significantly. Separate models were calculated and compared for mf-dyads and mm-dyads. Model details of the best models are provided. Best models and significant effects are highlighted in bold and effect directions are included.

**Additional file 6.** Statistical model comparisons and details of best models to explain the *number of conflicts* by the variables *species*, phylogeny (*clade*), forest type (*forest*) or the presence of reproductive females (*repro*). First, all models were compared to Base 0 model (Test 1, LRT_1_, P_1_-values). Second, the three alternative models were compared to the *species* model (Test 2, LRT_2_, P_2_-value). *Pair type* was added to the best model (#2) as an interaction term and improved the model significantly. Separate models were calculated and compared for mf-dyads and mm-dyads. Model details of the best models are provided. The best model and the significant effects are highlighted in bold and effect directions are included.

**Additional file 7.** Number of decided conflicts won by each male or female in each male–female or male–male dyad and resulting dominance relationships in all six species. Displayed are also the body mass differences between both dyad partners.

## Data Availability

The dataset generated and analysed during the current study is available from the corresponding author on reasonable request.

## Abbreviations

A: aggressive behavior
AIC: Akaike information criterion
BIC: Bayesian information criterion
DF: dry forest
FDD: female dominant dyads
HF: humid forest
max: maximum
IRS: Inter-River System
IUCN: International Union for Conservation of Nature
Mbon: *Microcebus bongolavensis*
Mdan: *Microcebus danfossi*
mf: male–female
min: minimum
mm: male–male
Mmam: *Microcebus mamiratra*
Mmar: *Microcebus margotmarshae*
Mmyo: *Microcebus myoxinus*
Mrav: *Microcebus ravelobensis*
N: northern clade
NW: northwestern clade
repro: reproduction model
S: submissive behavior
SD: standard deviation
SE: standard error
W: western clade
