# Supplementary material for: Sources of variation in social tolerance in mouse lemurs (Microcebus spp.)
Source: BMC Ecol. 2019 May 17;19:20. doi: 10.1186/s12898-019-0236-x (PMC6525410; doi:10.1186/s12898-019-0236-x)
Supplement: Supplementary file 1 — Additional file 1. Species, study site, forest type, latitude (South) and longitude (East) in decimal degree, clade, total number of individual males (M) and females (F) captured at each site, number of studied male–male (MM) and male–female (MF) dyads, study period and number of reproductively active females in observed dyads and in capture population. [file 12898_2019_236_MOESM1_ESM.docx]

Additional file 1. Species, study site, forest type, latitude (South) and longitude (East) in decimal degree, clade, total number of individual males (M) and females (F) captured at each site, number of studied male-male (MM) and male-female (MF) dyads, study period and number of reproductively active females in observed dyads and in capture population

| **Species** | **Site** | **FT** | **South** | **East** | **Clade** | **No. of ind.** | **Study dyads** | **Study period** | **Females in dyads** | **RA%** |
| --- | --- | --- | --- | --- | --- | --- | --- | --- | --- | --- |
| *M. myoxinus* | Bombetoka | DDF | -15.883° | 46.233° | 3 | 18M, 15F | 6MM, 6MF | 09.09. - 13.10.2015 | 6CL | 0 |
| *M. ravelobensis* | Ampijoroa | DDF | -16.317° | 46.817° | 2 | 15M, 25F | 6MM, 6MF | 30.04. - 14.06.2015 | 6CL | 0 |
| *M. bongolavensis* | Marosely | DDF | -15.650° | 47.583° | 2 | 16M, 19F | 5MM, 6MF | 09.07. - 13.08.2015 | 6CL | 0 |
| *M. danfossi* | Anjiamangirana | DDF | -15.150° | 47.730° | 2 | 22M, 23F | 6MM, 6MF | 17.09. - 22.10.2016 | 1CL, 2O, 2RC, 1P | 71.4 |
| *M. margotmarshae* | Ankaramibe | EHF | -13.964° | 48.198° | 1 | 18M, 21F | 6MM, 6MF | 09.08. - 13.09.2016 | 3CL, 2RC, 1L | 42.1 |
| *M. mamiratra* | Lokobe | EHF | -13.383° | 48.333° | 1 | 22M, 16F | 6MM, 6MF | 25.06. - 31.07.2016 | 3CL, 1S, 1RC, 1P | 46.2 |

FT= forest type; DDF= dry deciduous forest; EHF= evergreen humid forest; No. of ind= number of individuals captured at each site; CL: closed; S: swollen; O: estrus; RC: recent closely; P: pregnancy; L: lactation; RA% = percentage of reproductively active females among all captured females
