# Supplementary material for: Sources of variation in social tolerance in mouse lemurs (Microcebus spp.)
Source: BMC Ecol. 2019 May 17;19:20. doi: 10.1186/s12898-019-0236-x (PMC6525410; doi:10.1186/s12898-019-0236-x)
Supplement: Supplementary file 2 — Additional file 2. Statistical model comparisons and best model to explain the frequency of staying together in the sleeping box (SB) by the parameters species, phylogeny (clade), forest type (forest) or the presence of reproductive females (repro). First, all models were compared to Base 0 model (Test 1, LRT1, P1-values). Second, the three alternative models were compared to the species model (Test 2, LRT2, P2-value). Finally, pair type was added to the best model (#2) as an interaction term, but did not improve model fit. Model details for the best model are provided below. The best model is highlighted in bold and effect directions are included. [file 12898_2019_236_MOESM2_ESM.docx]

**Additional file 2** Statistical model comparisons and best model to explain the frequency of staying together in the sleeping box (SB) by the parameters species, phylogeny (clade), forest type (forest) or the presence of reproductive females (repro). First, all models were compared to Base 0 model (Test 1, LRT_1_, P_1_-values). Second, the three alternative models were compared to the species model (Test 2, LRT_2_, P_2_-value). Finally, *pair type* was added to the best model (#2) as an interaction term, but did not improve model fit. Model details for the best model are provided below. The best model is highlighted in bold and effect directions are included.

| **Model comparisons** | **df** | **AIC** | **BIC** | **logLiK** | **Test 1** | **LRT_1_** | **P_1_-value** | **Test 2** | **LRT_2_** | **P_2_-value** | **Effect** |
| --- | --- | --- | --- | --- | --- | --- | --- | --- | --- | --- | --- |
| #1 Base 0 | 2 | 751.0158 | 755.5412 | -373.5079 |  |  |  |  |  |  |  |
| **#2 Species** | **7** | **724.6198** | **740.4585** | **-355.3099** | **1 vs. 2** | **36.396** | **<0.0001** |  |  |  | **Mmyo > Mbon*, Mmar*, Mmam***/ Mmam < Mdan**, Mrav***** |
| #3 Forest | 3 | 736.1427 | 742.9307 | -365.0714 | 1 vs. 3 | 16.873 | <0.0001 | 2 vs. 3 | 19.523 | 6e-04 |  |
| #4 Clade | 4 | 732.6426 | 741.6933 | -362.3213 | 1 vs. 4 | 22.373 | <0.0001 | 2 vs. 4 | 14.023 | 0.0029 |  |
| #5 Repro | 3 | 743.2985 | 750.0865 | -368.6492 | 1 vs. 5 | 9.717 | 0.0018 | 2 vs. 5 | 26.679 | <0.0001 |  |
|  |  |  |  |  |  |  |  |  |  |  |  |
| #6 Species * pair type | 13 | 730.8097 | 760.2245 | -352.4048 |  |  |  | 2 vs. 6 | 5.810 | 0.4448 |  |
|  |  |  |  |  |  |  |  |  |  |  |  |
| **Best model: #2** |  | **Coefficient** | **SE** | **t-value** | **p-value** |  |  |  |  |  |  |
| (Intercept) |  | 154.70550 | 10.88210 | 14.216517 | <0.0001 |  |  |  |  |  |  |
| *M. ravelobensis* |  | -15.40638 | 15.38961 | -1.001090 | 0.3205 |  |  |  |  |  |  |
| *M. bongolavensis* |  | -53.60451 | 15.73549 | -3.406600 | **0.0011** |  |  |  |  |  |  |
| *M. danfossi* |  | -28.34859 | 15.38961 | -1.842061 | 0.0700 |  |  |  |  |  |  |
| *M. margotmarshae* |  | -47.07258 | 15.38961 | -3.058725 | **0.0032** |  |  |  |  |  |  |
| *M. mamiratra* |  | -90.98844 | 15.38961 | -5.912330 | **<0.0001** |  |  |  |  |  |  |

Mmyo: *M. myoxinus*, Mbon: *M. bongolavensis*, Mrav: *M. ravelobensis*, Mdan: *M. danfossi*, Mmar: *M. margotmarshae*, Mmam: *M. mamiratra*. *: p<0.05, **: p<0.01, ***: p<0.001
