# Supplementary material for: Sources of variation in social tolerance in mouse lemurs (Microcebus spp.)
Source: BMC Ecol. 2019 May 17;19:20. doi: 10.1186/s12898-019-0236-x (PMC6525410; doi:10.1186/s12898-019-0236-x)
Supplement: Supplementary file 4 — Additional file 4. Statistical model comparisons and details of best models to explain the frequency of total body contact by the parameters species, phylogeny (clade), forest type (forest) or the presence of reproductive females (repro). First, all models were compared to Base 0 model (Test 1, LRT1, P1-values), Pair type was added to the species model (#2) as an interaction term and improved the model significantly. Separate models were calculated and compared for mf-dyads and mm-dyads. Model details of the best models are provided. Best models and significant effects are highlighted in bold and effect directions are included. [file 12898_2019_236_MOESM4_ESM.docx]

**Additional file 4** Statistical model comparisons and details of best models to explain the frequency of *total body contact* by the parameters *species*, phylogeny (*clade*), forest type (*forest*) or the presence of reproductive females (*repro*). First, all models were compared to Base 0 model (Test 1, LRT_1_, P_1_-values), *Pair type* was added to the species model (#2) as an interaction term and improved the model significantly. Separate models were calculated and compared for mf-dyads and mm-dyads. Model details of the best models are provided. Best models and significant effects are highlighted in bold and effect directions are included.

| **Model comparisons - all** | **df** | **AIC** | **BIC** | **logLiK** | **Test 1** | **LRT_1_** | **P_1_-value** | **Test 2** | **LRT_2_** | **P_2_-value** | **Effect** |
| --- | --- | --- | --- | --- | --- | --- | --- | --- | --- | --- | --- |
| #1 Base 0 - all | 2 | 250.1359 | 254.6613 | -123.0680 |  |  |  |  |  |  |  |
| #2 Species- all | 7 | 249.1435 | 264.9823 | -117.5718 | 1 vs. 2 | 10.992 | 0.0515 |  |  |  |  |
| #3 Forest- all | 3 | 250.7117 | 257.4998 | -122.3559 | 1 vs. 3 | 1.424 | 0.2327 |  |  |  |  |
| #4 Clade- all | 4 | 252.3664 | 261.4171 | -122.1832 | 1 vs. 4 | 1.770 | 0.4128 |  |  |  |  |
| #5 Repro- all | 3 | 252.0998 | 258.8879 | -123.0499 | 1 vs. 5 | 0.036 | 0.8493 |  |  |  |  |
|  |  |  |  |  |  |  |  |  |  |  |  |
| **#6 Species * pair type** | **13** | **247.2564** | **276.6712** | **-110.6282** | **1 vs. 6** | **24.880** | **0.0095** |  |  |  |  |
|  |  |  |  |  |  |  |  |  |  |  |  |
| **Model comparisons - mf** |  |  |  |  |  |  |  |  |  |  |  |
| #7 Base 0 – mf | 2 | 134.4115 | 137.5785 | -65.2058 |  |  |  |  |  |  |  |
| **#8 Species – mf** | **7** | **132.6715** | **143.7561** | **-59.3358** | **7 vs. 8** | **11.740** | **0.0385** |  |  |  | **Mbon > Mmyo+, Mdan+** |
| #9 Forest – mf | 3 | 136.1564 | 140.9070 | -65.0782 | 7 vs. 9 | 0.255 | 0.6135 | **8 vs. 9** | **11.485** | **0.0216** |  |
| #10 Clade – mf | 4 | 135.6658 | 141.9999 | -63.8329 | 7 vs. 10 | 2.746 | 0.2534 | **8 vs. 10** | **8.994** | **0.0294** |  |
| #11 Repro – mf | 3 | 134.9527 | 139.7032 | -64.4763 | 7 vs. 11 | 1.459 | 0.2271 | **8 vs. 11** | **10.281** | **0.036** |  |
|  |  |  |  |  |  |  |  |  |  |  |  |
| **Best model – mf:** |  | **Coefficient** | **SE** | **t-value** | **p-value** |  |  |  |  |  |  |
| **#8 Species – mf** |  |  |  |  |  |  |  |  |  |  |  |
| (Intercept) |  | 0.7170901 | 0.5624562 | 1.2749262 | 0.2121 |  |  |  |  |  |  |
| *M. ravelobensis* |  | 0.6683908 | 0.7954332 | 0.8402854 | 0.4074 |  |  |  |  |  |  |
| *M. bongolavensis* |  | 2.3800346 | 0.7954332 | 2.9921240 | **0.0055** |  |  |  |  |  |  |
| *M. danfossi* |  | 0.1878942 | 0.7954332 | 0.2362162 | 0.8149 |  |  |  |  |  |  |
| *M. margotmarshae* |  | 0.6836015 | 0.7954332 | 0.8594079 | 0.3969 |  |  |  |  |  |  |
| *M. mamiratra* |  | 0.4068131 | 0.7954332 | 0.5114359 | 0.6128 |  |  |  |  |  |  |
|  |  |  |  |  |  |  |  |  |  |  |  |
| **Model comparisons - mm** | Df | **AIC** | **BIC** | **logLiK** | **Test 1** | **LRT_1_** | **P_1_-value** | **Test 2** | **LRT_2_** | **P_2_-value** | **Effect** |
| #12 Base 0 – mm | 2 | 115.5961 | 118.7068 | -55.79804 |  |  |  |  |  |  |  |
| #13 Species – mm | 7 | 115.1273 | 126.0147 | -50.56363 | 12 vs. 13 | 10.469 | 0.063 |  |  |  |  |
| **#14 Forest – mm** | **3** | **110.4507** | **115.1167** | **-52.22535** | **12 vs. 14** | **7.145** | **0.0075** |  |  |  | **Humid > dry**** |
| **#15 Clade – mm** | **4** | **111.2424** | **117.4638** | **-51.62122** | **12 vs. 15** | **8.354** | **0.0153** | **14 vs. 15** | **1.208** | **0.2717** | **N > NW **** |
| #16 Repro – mm | 3 | 116.2248 | 120.8908 | -55.11239 | 12 vs. 16 | 1.371 | 0.2416 |  |  |  |  |
|  |  |  |  |  |  |  |  |  |  |  |  |
| **Best models – mm:** |  | **Coefficient** | **SE** | **t-value** | **p-value** |  |  |  |  |  |  |
| **#14 Forest - mm** |  |  |  |  |  |  |  |  |  |  |  |
| (Intercept) |  | 1.575111 | 0.2310520 | 6.817130 | 0.00 |  |  |  |  |  |  |
| Forest-humid |  | 1.078776 | 0.3945964 | 2.733872 | **0.01** |  |  |  |  |  |  |
|  |  |  |  |  |  |  |  |  |  |  |  |
| **#15 Clade - mm** |  |  |  |  |  |  |  |  |  |  |  |
| (Intercept) |  | 2.653888 | 0.3192777 | 8.312159 | 0.0000 |  |  |  |  |  |  |
| Clade-nw |  | -1.224030 | 0.4170068 | -2.935275 | **0.0061** |  |  |  |  |  |  |
| Clade-w |  | -0.667224 | 0.5530053 | -1.206542 | 0.2365 |  |  |  |  |  |  |

Mmyo: *M. myoxinus*, Mbon: *M. bongolavensis*, Mrav: *M. ravelobensis*, Mdan: *M. danfossi*, Mmar: *M. margotmarshae*, Mmam: *M. mamiratra*. +: p<0.1, *: p<0.05, **: p<0.01, ***: p<0.001
