# Supplementary material for: Sources of variation in social tolerance in mouse lemurs (Microcebus spp.)
Source: BMC Ecol. 2019 May 17;19:20. doi: 10.1186/s12898-019-0236-x (PMC6525410; doi:10.1186/s12898-019-0236-x)
Supplement: Supplementary file 5 — Additional file 5. Statistical model comparisons and details of best models to explain aggression rate by the parameters species, phylogeny (clade), forest type (forest) or the presence of reproductively active females (repro). First, all models were compared to Base 0 model (Test 1, LRT1, P1-values), Pair type and sex were added to the models #2–#4 and improved them significantly. Separate models were calculated and compared for mf-dyads and mm-dyads. Model details of the best models are provided. Best models and significant effects are highlighted in bold and effect directions are included. [file 12898_2019_236_MOESM5_ESM.docx]

**Additional file 5** Statistical model comparisons and details of best models to explain *aggression rate* by the parameters *species*, phylogeny (*clade*), forest type (*forest*) or the presence of reproductively active females (*repro*). First, all models were compared to Base 0 model (Test 1, LRT_1_, P_1_-values), *Pair type* and *sex* were added to the models #2 - #4 and improved them significantly. Separate models were calculated and compared for mf-dyads and mm-dyads. Model details of the best models are provided. Best models and significant effects are highlighted in bold and effect directions are included.

| **Model comparisons - all** | **df** | **AIC** | **BIC** | **logLiK** | **Test 1** | **LRT_1_** | **P_1_-value** | **Test 2** | **LRT_2_** | **P_2_-value** | **Effect** |
| --- | --- | --- | --- | --- | --- | --- | --- | --- | --- | --- | --- |
| #1 Base 0 - all | 3 | 300.0916 | 308.9590 | -147.0458 |  |  |  |  |  |  |  |
| **#2 Species- all** | **8** | **276.2015** | **299.8481** | **-130.1007** | **1 vs. 2** | **33.890** | **<.0001** |  |  |  |  |
| **#3 Forest- all** | **4** | **273.0640** | **284.8873** | **-132.5320** | **1 vs. 3** | **29.028** | **<.0001** |  |  |  |  |
| **#4 Clade- all** | **5** | **274.3016** | **289.0807** | **-132.1508** | **1 vs. 4** | **29.790** | **<.0001** |  |  |  |  |
| #5 Repro- all | 4 | 298.9368 | 310.7602 | -145.468 | 1 vs. 5 | 3.155 | 0.0757 |  |  |  |  |
|  |  |  |  |  |  |  |  |  |  |  |  |
| **#6 Species + Type + Sex** | **10** | **256.1406** | **285.6988** | **-118.0703** |  |  |  | **2 vs. 6** | **24.061** | **<.0001** |  |
| **#7 Forest + Type + Sex** | **6** | **253.3205** | **271.0555** | **-120.6603** |  |  |  | **3 vs. 7** | **23.744** | **<.0001** |  |
| **#8 Clade + Type + Sex** | **7** | **254.5512** | **275.2420** | **-120.2756** |  |  |  | **4 vs. 8** | **23.750** | **<.0001** |  |
|  |  |  |  |  |  |  |  |  |  |  |  |
| **Model comparisons - mf** |  |  |  |  |  |  |  |  |  |  |  |
| #9 Base 0 - mf | 3 | 173.7870 | 180.6170 | -83.89351 |  |  |  |  |  |  |  |
| **#10 Species + Sex** | **9** | **127.8671** | **148.3571** | **-54.93356** | **9 vs. 10** | **57.920** | **<.0001** |  |  |  | **Mmam, Mmar > Mmyo***, Mrav***^,^*, Mbon***, Mdan***^,^*** |
| **#11 Forest + Sex** | **5** | **129.9441** | **141.3274** | **-59.97203** | **9 vs. 11** | **47.843** | **<.0001** | **10 vs.11** | **10.077** | **0.0392** |  |
| **#12 Clade + Sex** | **6** | **131.6623** | **145.3223** | **-59.83113** | **9 vs. 12** | **48.125** | **<.0001** | **10 vs. 12** | **9.795** | **0.0204** |  |
|  |  |  |  |  |  |  |  |  |  |  |  |
| **Best model – mf:** |  | **Coefficient** | **SE** | **t-value** | **p-value** |  |  |  |  |  |  |
| **#10 Species + Sex** |  |  |  |  |  |  |  |  |  |  |  |
| (Intercept) |  | 0.6630278 | 0.1702971 | 3.893361 | 0.0004 |  |  |  |  |  |  |
| *M. ravelobensis* |  | 0.2335494 | 0.2229712 | 1.047442 | 0.3033 |  |  |  |  |  |  |
| *M. bongolavensis* |  | -0.1263996 | 0.2229712 | -0.566888 | 0.5750 |  |  |  |  |  |  |
| *M. danfossi* |  | 0.2062540 | 0.2229712 | 0.925025 | 0.3623 |  |  |  |  |  |  |
| *M. margotmarshae* |  | 0.8758513 | 0.2229712 | 3.928092 | **0.0005** |  |  |  |  |  |  |
| *M. mamiratra* |  | 1.4556179 | 0.2229712 | 6.528278 | **0.0000** |  |  |  |  |  |  |
| Sex-m |  | -0.5287337 | 0.1287325 | -4.107228 | **0.0002** |  |  |  |  |  | **M < F** |
|  |  |  |  |  |  |  |  |  |  |  |  |
| **Model comparisons - mm** | Df | **AIC** | **BIC** | **logLiK** | **Test 1** | **LRT_1_** | **P_1_-value** | **Test 2** | **LRT_2_** | **P_2_-value** | **Effect** |
| #13 Base 0 – mm |  | 120.7162 | 127.4617 | -57.35808 |  |  |  |  |  |  |  |
| #14 Species – mm |  | 123.9536 | 141.9416 | -53.97679 | 13 vs. 14 | 6.763 | 0.2389 |  |  |  |  |
| **#15 Forest – mm** |  | **117.7190** | **126.7130** | **-54.85949** | **13 vs. 15** | **4.997** | **0.0254** |  |  |  | **Humid > dry*** |
| #16 Clade – mm |  | 119.0803 | 130.3228 | -54.54014 | 13 vs. 16 | 5.636 | 0.0597 |  |  |  |  |
|  |  |  |  |  |  |  |  |  |  |  |  |
| **Best model – mm:** |  | **Coefficient** | **SE** | **t-value** | **p-value** |  |  |  |  |  |  |
| **#15 Forest - mm** |  |  |  |  |  |  |  |  |  |  |  |
| (Intercept) |  | 0.4786832 | 0.1040305 | 4.601374 | 0.0001 |  |  |  |  |  |  |
| Forest-humid |  | 0.4058431 | 0.1776659 | 2.284305 | **0.0289** |  |  |  |  |  |  |
|  |  |  |  |  |  |  |  |  |  |  |  |

Mmyo: *M. myoxinus*, Mbon: *M. bongolavensis*, Mrav: *M. ravelobensis*, Mdan: *M. danfossi*, Mmar: *M. margotmarshae*, Mmam: *M. mamiratra*. *: p<0.05, **: p<0.01, ***: p<0.001, if two significance levels are provided, the first refers to the first compared species and the second refers to the second compared species.
