# Supplementary material for: Sources of variation in social tolerance in mouse lemurs (Microcebus spp.)
Source: BMC Ecol. 2019 May 17;19:20. doi: 10.1186/s12898-019-0236-x (PMC6525410; doi:10.1186/s12898-019-0236-x)
Supplement: Supplementary file 7 — Additional file 7. Number of decided conflicts won by each male or female in each male–female or male–male dyad and resulting dominance relationships in all six species. Displayed are also the body mass differences between both dyad partners. [file 12898_2019_236_MOESM7_ESM.docx]

**Additional file 7** Number of decided conflicts won by each male or female in each male-female or male-male dyad and resulting dominance relationships in all six species

| Dyad type | Species | Dyad no. | No. of decided conflicts | Ind.1 | Ind.2ª | *p-value* | Dominance | Mass difference^b^ (g) |
| --- | --- | --- | --- | --- | --- | --- | --- | --- |
| Male-female | *M. myoxinus* | 1 | 2 | 1 | 1 | 0.5 |  | +1 |
|  | *M. myoxinus* | 2 | 2 | 2 | 0 | 0.239 |  | -1 |
|  | *M. myoxinus* | 3 | 3 | 0 | 3 | 0.124 |  | +1 |
|  | *M. myoxinus* | 4 | 1 | 1 | 0 | 0.5 |  | -1 |
|  | *M. myoxinus* | 5 | 1 | 0 | 1 | 0.5 |  | +7 |
|  | *M. myoxinus* | 6 | 1 | 0 | 1 | 0.5 |  | 0 |
|  | *M. ravelobensis* | 1 | 0 | 0 | 0 |  |  | 0 |
|  | *M. ravelobensis* | 4 | 14 | 0 | 14 | **<0.001** | FD | -2 |
|  | *M. ravelobensis* | 5 | 0 | 0 | 0 |  |  | +3 |
|  | *M. ravelobensis* | 6 | 5 | 2 | 3 | 0.5 |  | -1 |
|  | *M. ravelobensis* | 7 | 2 | 1 | 1 | 0.5 |  | -6 |
|  | *M. ravelobensis* | 8 | 11 | 2 | 9 | **0.035** | FD | +5 |
|  | *M. bongolavensis* | 1 | 0 | 0 | 0 |  |  | +1 |
|  | *M. bongolavensis* | 2 | 0 | 0 | 0 |  |  | 0 |
|  | *M. bongolavensis* | 3 | 4 | 0 | 4 | 0.066 |  | -9 |
|  | *M. bongolavensis* | 4 | 4 | 4 | 0 | 0.066 |  | -9 |
|  | *M. bongolavensis* | 5 | 8 | 0 | 8 | **0.007** | FD | +4 |
|  | *M. bongolavensis* | 6 | 4 | 0 | 4 | 0.066 |  | -2 |
|  | *M. danfossi* | 1 | 29 | 4 | 25 | **<0.001** | FD | -8 |
|  | *M. danfossi* | 2 | 1 | 0 | 1 | 0.5 |  | +4 |
|  | *M. danfossi* | 3 | 43 | 1 | 41 | **<0.001** | FD | 0 |
|  | *M. danfossi* | 4 | 1 | 0 | 1 | 0.5 |  | +12 |
|  | *M. danfossi* | 5 | 18 | 3 | 15 | **0.004** | FD | -2 |
|  | *M. danfossi* | 6 | 1 | 0 | 1 | 0.5 |  | -6 |
|  | *M. margotmarshae* | 1 | 22 | 16 | 6 | **0.028** | MD | -2 |
|  | *M. margotmarshae* | 2 | 3 | 1 | 2 | 0.5 |  | -12 |
|  | *M. margotmarshae* | 4 | 25 | 0 | 25 | **<0.001** | FD | +12 |
|  | *M. margotmarshae* | 5 | 144 | 2 | 142 | **<0.001** | FD | -1 |
|  | *M. margotmarshae* | 6 | 1 | 0 | 1 | 0.5 |  | +8 |
|  | *M. margotmarshae* | 7 | 30 | 9 | 21 | **0.022** | FD | -11 |
|  | *M. mamiratra* | 1 | 201 | 5 | 196 | **<0.001** | FD | -14 |
|  | *M. mamiratra* | 2 | 47 | 1 | 46 | **<0.001** | FD | +2 |
|  | *M. mamiratra* | 3 | 51 | 3 | 48 | **<0.001** | FD | +2 |
|  | *M. mamiratra* | 4 | 85 | 4 | 81 | **<0.001** | FD | 0 |
|  | *M. mamiratra* | 5 | 302 | 4 | 298 | **<0.001** | FD | -6 |
|  | *M. mamiratra* | 6 | 131 | 10 | 121 | **<0.001** | FD | +3 |
| Male1-male2 | *M. myoxinus* | 7 | 0 | 0 | 0 |  |  | 0 |
|  | *M. myoxinus* | 8 | 0 | 0 | 0 |  |  | -2 |
|  | *M. myoxinus* | 9 | 0 | 0 | 0 |  |  | -2 |
|  | *M. myoxinus* | 10 | 1 | 1 | 0 | 0.5 |  | +14 |
|  | *M. myoxinus* | 11 | 2 | 1 | 1 | 0.5 |  | +4 |
|  | *M. myoxinus* | 12 | 0 | 0 | 0 |  |  | -1 |
|  | *M. ravelobensis* | 2 | 12 | 9 | 3 | 0.074 |  | -25 |
|  | *M. ravelobensis* | 3 | 4 | 3 | 1 | 0.5 |  | +10 |
|  | *M. ravelobensis* | 9 | 5 | 2 | 3 | 0.5 |  | -5 |
|  | *M. ravelobensis* | 10 | 4 | 4 | 0 | 0.066 |  | +5 |
|  | *M. ravelobensis* | 11 | 0 | 0 | 0 |  |  | -2 |
|  | *M. ravelobensis* | 12 | 0 | 0 | 0 |  |  | 0 |
|  | *M. bongolavensis* | 7 | 18 | 3 | 15 | **0.004** | M2 | -10 |
|  | *M. bongolavensis* | 8 | 10 | 9 | 1 | **0.013** | M1 | -4 |
|  | *M. bongolavensis* | 9 | 6 | 0 | 6 | **0.02** | M2 | -1 |
|  | *M. bongolavensis* | 10 | 0 | 0 | 0 |  |  | -20 |
|  | *M. bongolavensis* | 11 | 28 | 19 | 9 | **0.044** | M1 | -17 |
|  | *M. danfossi* | 7 | 21 | 6 | 15 | **0.040** | M2 | +1 |
|  | *M. danfossi* | 8 | 1 | 1 | 0 | 0,5 |  | +1 |
|  | *M. danfossi* | 9 | 15 | 3 | 12 | **0.019** | M2 | 0 |
|  | *M. danfossi* | 10 | 15 | 10 | 5 | 0.151 |  | -2 |
|  | *M. danfossi* | 11 | 0 | 0 | 0 |  |  | -1 |
|  | *M. danfossi* | 12 | 1 | 0 | 1 | 0.5 |  | +1 |
|  | *M. margotmarshae* | 3 | 10 | 1 | 9 | **0.013** | M2 | +6 |
|  | *M. margotmarshae* | 8 | 1 | 0 | 1 | 0.5 |  | -4 |
|  | *M. margotmarshae* | 9 | 56 | 46 | 10 | **<0.001** | M1 | +5 |
|  | *M. margotmarshae* | 10 | 2 | 1 | 1 | 0.5 |  | -3 |
|  | *M. margotmarshae* | 11 | 8 | 8 | 0 | **0.006** | M1 | 0 |
|  | *M. margotmarshae* | 12 | 7 | 5 | 2 | 0.5 |  | -2 |
|  | *M. mamiratra* | 7 | 79 | 2 | 77 | **<0.001** | M2 | +4 |
|  | *M. mamiratra* | 8 | 0 | 0 | 0 |  |  | +1 |
|  | *M. mamiratra* | 9 | 63 | 9 | 54 | **<0.001** | M2 | -15 |
|  | *M. mamiratra* | 10 | 8 | 5 | 3 | 0.361 |  | +1 |
|  | *M. mamiratra* | 11 | 15 | 13 | 2 | **0.005** | M1 | +16 |
|  | *M. mamiratra* | 12 | 18 | 13 | 5 | **0.049** | M1 | +4 |

Ind.: number of conflicts won by each individual of the respective dyad, ^a^: In male-female dyads individual 2 is always the female, ^b^: Negative value: Ind. 1 lighter than Ind. 2 - positive value: Ind. 1 heavier than Ind. 2, p-value: result of Binomial test, Bold values: p < 0.05, FD = female dominance; MD = male dominance; M1 = male 1 dominant; M2 = male 2 dominant
